# A comparative analysis of microbial profile of Guinea fowl and chicken using metagenomic approach

**DOI:** 10.1371/journal.pone.0191029

**Published:** 2018-03-01

**Authors:** Sarayu Bhogoju, Samuel Nahashon, Xiaofei Wang, Carl Darris, Agnes Kilonzo-Nthenge

**Affiliations:** 1 Department of Agricultural and Environmental Sciences, Tennessee State University, Nashville, Tennessee, United States of America; 2 Department of Biological Sciences, Tennessee State University, Nashville, Tennessee, United States of America; 3 Department of Nephrology, Vanderbilt University, Nashville, Tennessee, United States of America; 4 Department of Human Sciences, Tennessee State University, Nashville, Tennessee, United States of America; Sun Yat-Sen University, CHINA

## Abstract

Probiotics are live microbial feed supplements that promote growth and health to the host by minimizing non-essential and pathogenic microorganisms in the host’s gastrointestinal tract (GIT). The campaign to minimize excessive use of antibiotics in poultry production has necessitated development of probiotics with broad application in multiple poultry species. Design of such probiotics requires understanding of the diversity or similarity in microbial profiles among avian species of economic importance. Therefore, the objective of this research was to establish and compare the microbial profiles of the GIT of Guinea fowl and chicken and to establish the microbial diversity or similarity between the two avian species. A metagenomic approach consisting of the amplification and sequence analysis of the hypervariable regions V1-V9 of the 16S rRNA gene was used to identify the GIT microbes. Collectively, we detected more than 150 microbial families. The total number of microbial species detected in the chicken GIT was higher than that found in the Guinea Fowl GIT. Our studies also revealed phylogenetic diversity among the microbial species found in chicken and guinea fowl. The phylum *Firmicutes* was most abundant in both avian species whereas Phylum *Actinobacteria* was most abundant in chickens than Guinea fowls. The diversity of the microbial profiles found in broiler chickens and Guinea fowls suggest that the design of effective avian probiotics would require species specificity.

## Introduction

The increased demand for poultry and poultry products has contributed to attempts to raise poultry in confinements and in large numbers and smaller floor space. This predisposes birds to stress and vulnerability to poultry diseases, especially those caused by bacterial infections. To counter this, the industry employs antibiotics at therapeutic doses to prevent disease outbreak, increase efficiency of feed utilization and growth performance. Antibiotics are also used in food animal production to treat clinically sick animals and to prevent or reduce the incidence of infectious diseases. The use of low doses of antibiotics is the primary cause of antimicrobial drug resistant strains of pathogenic bacteria. Such resistance can be transferred to the consumer and create resistance to common antibiotics treating human infections. This is of great concern to the poultry industry and the consumer alike. There is therefore concerted effort to reduce the use of antibiotics in the poultry industry, inviting the use of alternatives to antibiotics.

In the recent past, nutritionists and veterinary experts have paid keen attention on proper utilization of nutrients and the use of probiotics for growth promotion of poultry. In broiler nutrition, probiotic species belonging to *Lactobacillus*, *Streptococcus*, *Bacillus*, *Bifidobacterium*, *Enterococcus*, *Aspergillus*, *Candida*, and *Saccharomyces* tend to have beneficial effect on broiler performance [1], which includes modulation of intestinal microflora, pathogen inhibition, intestinal histological changes, immunomodulation, certain haemato-biochemical parameters, improving sensory characteristics of dressed broiler meat [2] and promoting microbiological meat quality of broilers. Anticipated mechanisms of pathogen inhibition by the probiotic microorganisms include competition for nutrients, production of antimicrobial conditions, antimicrobial compounds (volatile fatty acids, and bacteriocin), lowering GIT pH, and competition for binding sites on the intestinal epithelium and stimulation of the immune system [3]. Consequently, most of these proposed modes of action of probiotics have not been researched thoroughly leading to paucity of knowledge in this area; hence, there is a dire need for clear understanding of the modes of action of probiotics.

The quest for alternatives to antibiotics has also been matched by the demand for alternative poultry species such as the Guinea fowl. Efforts are underway to improve production efficiency of the Guinea fowl under similar management conditions to chickens. Commercialization of Guinea fowl production for meat and eggs has progressed in the United States, Australia and around the world [4]. The guinea fowl has also been gaining popularity in the United States and Europe as a delicacy owing to its lean meat, high protein content, unique flavor [5] and resistance to diseases [6]. These are the two main species researched and reared at Tennessee State University [7, 8, 9, and 10]. The microbial profiles of other species such as ducks and turkeys will also be evaluated in the near future. Better still, this would allow design of probiotics that have broad application to these multiple avian species. The primary premise is to ensure that the population of beneficial microorganisms is maintained or increased while minimizing the population of non-essential and pathogenic microorganisms. The other advantage of revealing microorganisms in the GIT of these birds is to allow harvesting of the beneficial microorganisms from the host for developing the probiotics since they already can thrive under the GIT environment of the host.

Thus when seeking effective probiotics for chickens other avian species, such as the Guinea fowl, must be put into consideration. While efforts to establish beneficial effect and modes of action of probiotics in poultry have focused on chickens, very limited effort has been directed to developing effective probiotics for other avian species such as the Guinea fowl. To determine whether or not probiotics can be effective in conferring beneficial effects across avian species, the microbial profiles of the gastrointestinal tract of these species must be established as well.

In this report, the microbial profiles of the GIT of the chicken and Guinea fowl were evaluated using the metagenomics approach to resolve the microbial diversity of the two avian species. Metagenomics is a culture independent method that utilizes DNA sequencing techniques to study DNA extracted directly from environmental samples is employed. In this study, DNA was extracted from the GIT environment of the chicken and Guinea fowl. The hypervariable region encoding 16s rRNA is often targeted using the metagenomics approach to reveal the composition of microbial populations in organisms [11]. The 9 variable regions (V1-V9) of the 16s rRNA bears significant degrees of sequence diversity and has been reported to effectively distinguish maximally the bacterial communities within the gastro intestinal tract of various organisms [12]. Technological advances such as the next generation sequencing platform provides an opportunity to evaluate the host specific microbial diversity of GIT with 16s metagenomics approach [13]. The objective of this study was therefore to establish and compare the microbial profile of GIT of chicken and Guinea fowl and to establish the microbial diversity or similarity between these two avian species. The outcome would guide in determining whether or not these two avian species would require customization of probiotics. An additional objective was to reveal new microbes within the GIT of these two avian species which may also be utilized in developing probiotics in the future.

## Materials and methods

All animal studies adhered to the institutional animal care and use committee’s (IACUC) guidelines and were approved by IACUC. Ten chickens and ten Guinea fowls (20 weeks old) were sampled from poultry flocks raised at the Tennessee State University poultry research farm. The broiler chickens were derived from the same parental line and the guinea fowl were of the French variety derived also from same parental line. The birds were raised using standard management techniques [14] and were fed isocaloric and isonitrogenous (3,150 ME Kcal ME/kg diet and 21% CP, respectively) diets for 20 weeks. The mash feed and water did not contain probiotics and were provided at free choice. Both chickens and Guinea fowls were sacrificed by cervical dislocation and whole GIT content was collected with sterile forceps and a sterile knife (depending on consistency of the digesta). The GIT contents and the lining of intestinal epithelium were scraped into a sterile 50 mL polystyrene tube containing 30 mL of sterile 1xPBS solution [15]. The lining of the epithelium was scraped to capture the microbes which were attached to the GIT epithelium. The diluted samples of GIT contents were placed in ice and immediately used for DNA extraction or stores at -80 ^o^C until use. DNA was extracted using the pure link Genomic DNA mini kit (Life Technologies, Waltham, MA, USA) protocol and the concentration of DNA was measured using a NanoDrop spectrophotometer (Thermo Fisher, Waltham, MA, USA) and agarose gel electrophoresis. From the quantification results the high quality DNA samples (A_260_/A_280_ = 1.85–1.90) of 10 chicken and 10 Guinea fowl, 5 DNA samples were pooled to make two separate pooled samples each of chickens and Guinea fowls and these pooled samples were used further for DNA library construction and NGS.

The 16s DNA library was constructed by following the instructions from 16s metagenomic kit and ion plus fragment library kit (Life Technologies). Primers were provided in the kit to amplify the 16s region of the DNA samples. Two sets of primers, respectively were designed to amplify V2-4-8 regions and V3-6, 7–9 regions of 16s gene. PCR amplicons of equal volume and concentration from the two primers were pooled and used in library construction. The quality of the constructed library was analyzed using Agilent 2100 Bio-analyzer (Agilent Technologies, Santa Clara, CA, USA) following the protocol of Agilent DNA7500 kit reagents (Agilent Technologies) and the library concentration was diluted up to 26–30 pM using nuclease free water. The diluted 16s library was used for template preparation using IonOneTouch-2 system by following instructions from ion PGM Template OT2 400 kit. The DNA template quality was analyzed using the qubit 2.0 flouorometer following instructions from IonOT2 400 kit using Ion sphere quality control kit (Life technologies). DNA quality scores were ensured for each of the two birds selected for the NGS.

The DNA template was enriched using Ion one Touch ES system (life technologies) following instructions from IonOT2 400 kit. After the enrichment DNA library samples were processed for amplification reaction using the protocol from Ion PGM 400 sequencing kit (Life technologies). The amplified sample was loaded onto 316V2 (life technologies) chip for sequencing using the Ion Torrent PGM system following instructions from Ion PGM 400 sequencing kit. The sequencing took about 6–7 h and was monitored through the ion torrent server. The detailed laboratory protocols can be accessed at http://dx.doi.org/10.17504/protocols.10.mdgc23w. Sequencing data were analyzed using the ion reporter software provided by Life Technologies. The Ion reporter software comprises a bundle of bioinformatics tools that aid streamlining and simplifying the analysis of Ion PGM sequencing data. The 16s metagenomic workflow in ion reporter was based on Core QIIME pipeline and the GreenGenes and Microseq ID databases for phylogenetic diversity. In QIIME pipeline, first stage involved clustering of all sequences from all test samples into Operational Taxonomic Units (OTUs) based on their sequence similarity. The OTUs in QIIME represented some degree of taxonomic relatedness. In stage two, QIIME was used to pick representative sequences from each OTU for downstream analysis. These representative sequences were used for taxonomic identification of the OTUs and phylogenetic alignment. Then the QIIME used the OTU file created above and to extract representative sequences from the fasta file by one of several methods and in Stage three, provided information on the microbial lineages found in microbial samples. By default, QIIME used the Ribosomal Database Project (RDP) classifier to assign taxonomic data to each representative sequence from stage two as described in Kuczynski et al. [16]. Thereafter the module classified individual reads from the sequencing data by using three reference library options; one being the Basic Local Alignment Search Tool (BLAST) to the curated GreenGenes database, secondly by BLAST alignment to the premium curated MicroSEQ ID database and thirdly by optimal two step BLAST alignment to both reference libraries. The alignment at various taxonomical levels followed the clinical and laboratory standards institute (CLSI) guidelines requiring the family, genus and species level to have <97%, >97% and >99% identity, respectively.

## Results and discussion

### 1. Libraries

Two representative pools of each of 5 chickens and 5 Guinea fowl 16s metagenomic libraries were used for sequencing with Ion PGM system. The sequencing runs obtained 5,798,290 and 4,296,772 reads for chicken and Guinea fowl respectively. Among the total reads, valid reads accounted for 68.7% and 57.2%, respectively, of the chicken and the Guinea fowl sequences (Table 1).

**Table 1:** Reads in chicken and Guinea fowl libraries.

Libraries	Total	Valid	Mapped
Chicken 1	2,678,815	2,116,221	1,067,244
Chicken 2	3,119,475	1,975,120	1,028,345
Chicken 1&2	5,798,290	3,980,729	2,204,484
% of reads	100	68.7	38
Guinea fowl 1	1,756,309	550,717	67,026
Guinea fowl 2	2,540,463	1,907,324	946,606
Guinea fowl 1&2	4,296,772	2,458,041	1,024,845
% of reads	100	57.2	23.9

**Fig 1:**
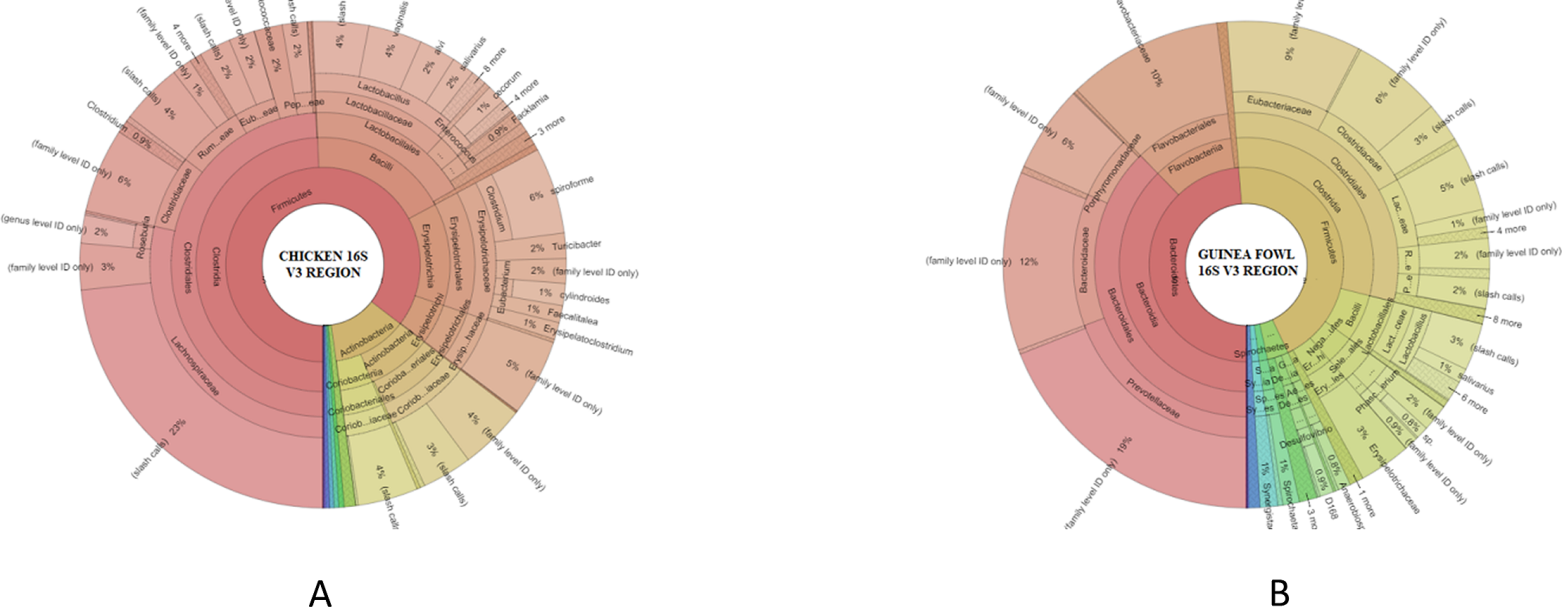
Phylogenetic composition of bacteria taxonomical levels derived from sequencing of V3 region of the 16s rRNA gene of a metagenomic library of chicken (1a) and Guinea fowl (1b) GIT contents. The percent of reads belonging to the bacterial taxonomical units from the chicken GIT microbiome is shown.

As the 16s rRNA gene consists of 9 variable regions which are useful in identifying specific phylogenetic diversity, the reads to particular variable regions was evaluated for the chicken and Guinea fowl profiles (Table 2). The V3 region is highly conserved region in 16s rRNA and as such maximum phylogenetic diversity was observed in the V3 region reads of both avian species (Fig 1). The V3 region had the majority reads, on average accounting for about 60% and 58% of the profiles of chicken and Guinea fowl libraries, respectively. In both chicken and Guinea fowl the distribution of microbiota is different (Fig 1A and 1B, respectively). Reads mapped to V7 and V8 accounted for 14% each in the chicken libraries, and 12% and 8%, respectively, of mapped reads in the Guinea fowl libraries. A major difference between the Guinea fowl and the chicken libraries came from V8 and V9 regions, where reads mapped to V8 accounted for only 14 and 8% of mapped reads in chicken and guinea fowl, respectively. On the other hand, reads mapped to V9 on average accounted for 1.3 and 18% in mapped reads of chicken and guinea fowl libraries, respectively. The V4 region accounted for 8.6 and 5.4% of all mapped reads in the chicken and guinea fowl libraries, respectively. The V2 region had the least number of mapped reads, which accounted for only 2.3 and 1.2% of reads in the chicken and guinea fowl libraries, respectively.

**Table 2:** Mapped reads in different variable regions.

Libraries		V2	V3	V4	V7	V8	V9
Chicken 1	Mapped	24,453	639,464	93,853	14,9349	14,6363	13,762
	% *	2.29	59.92	8.79	13.99	13.71	1.29
Chicken 2	Mapped	23,284	623,301	86,903	14,3692	13,8143	13,022
	%	2.26	60.61	8.45	13.97	13.43	1.27
Guinea fowl 1	Mapped	89	49,158	137	4059	27	13,556
	%	0.13	73.34	0.2	6.06	0.04	20.22
Guinea fowl 2	Mapped	11,736	418,957	52,208	16,5897	15,1232	14,6576
	%	1.24	44.26	5.52	17.53	15.98	15.48

Note

* mapped reads within the region over all mapped reads in that library.

### 2. Microbial phylogeny

**Fig 2:**
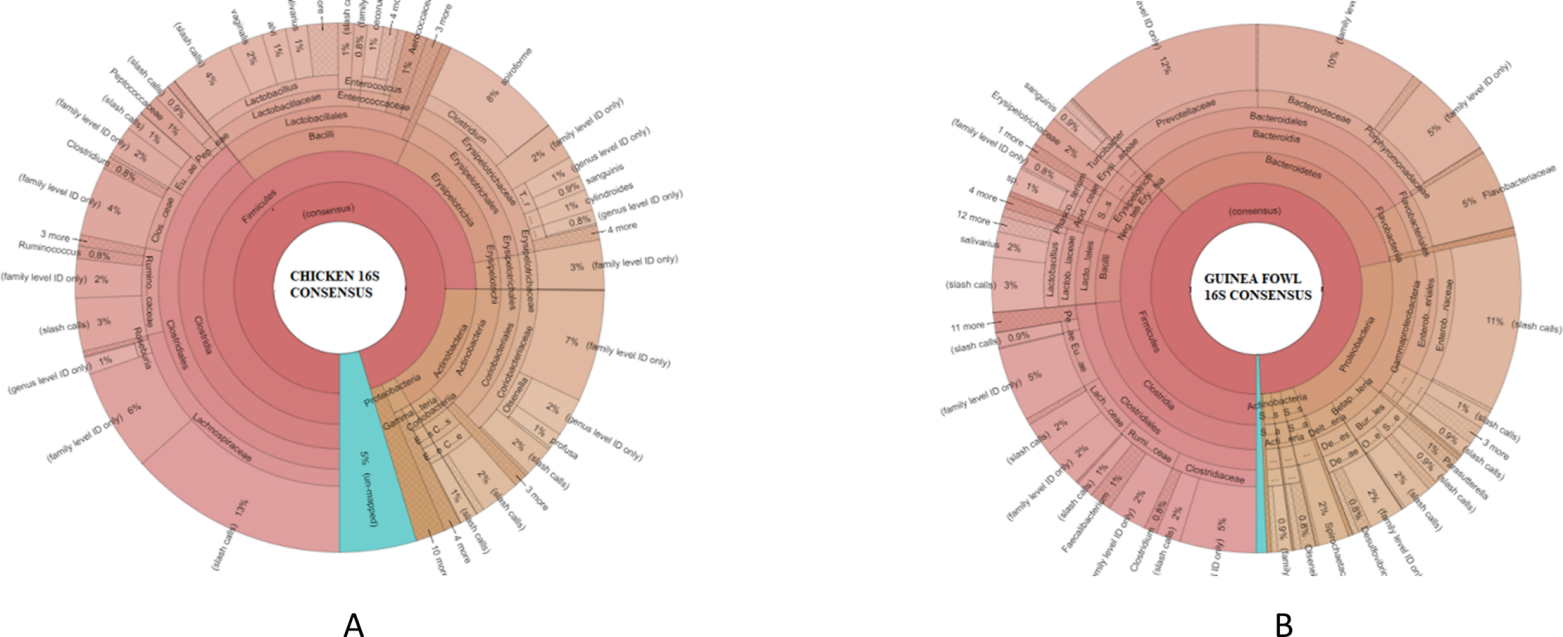
Consensus phylogenetic composition of bacteria taxonomical levels (phylum to species) derived from sequencing the 16s rRNA gene of a metagenomics library of chicken (a) and Guinea fowl (b) GIT contents. The percent of reads belonging to the bacterial taxonomical units from the chicken GIT microbiome is shown.

The distribution of total gut phyla of chicken and Guinea fowl is shown in Fig 2A and 2B, respectively. In total, 14 phyla were identified in the profiles of chicken and Guinea fowl combined. Those include, *Proteobacteria*, *Bacteroidetes*, *Firmicutes*, *Actinobacteria*, *Spirochaetes*, *Deferribacteres*, *Chloroflexi*, *Tenericutes*, *Fusobacteria*, *Cyanobacteria*, *Verrucomicrobia*, *Synergistetes*, *Thermotogae*, and *Lentisphaerae*. The Guinea fowl microbial profile showed the existence of *Verrucomicrobia* and *Lentisphaerae* where as these specific phyla were not found in chickens. Conversely, the chicken microbial profile showed *Fusobacteria* and *Thermotogae* that were not found in Guinea fowls. Among all these phyla, *Firmicutes* was the most dominated phylum in both chicken and Guinea fowl microbial profiles, which accounted for 79% and 43%, respectively. In chickens, the second most abundant phylum was *Actinobacteria* (17%), followed by *Proteobactria* (2%). In Guinea fowls, the second most abundant phylum after firmicutes was *Bacteroides* (29%), followed by *Proteobacteria* (23%). The phylum profiles were not much different from studies reported by others for avian hosts [17, 18].

#### 2.1 Family profile

There were 116 and 115 bacterial families found in the chicken and Guinea fowl libraries, respectively (S1 Table). Despite the diverse families of bacteria found in the gut, the most abundant 10 families (constituting 1% or more) accounted for 91% of the chicken enteric flora. The *Lachnospiraceae* family appeared most abundant in the chicken libraries, accounting for 23% of the GIT flora. Under some circumstances, this family may constitute up to 40% or more of the microbiota [19]. Family *Lachnospiraceae* belongs to the phylum *Firmicutes* and class *Clostridia*. These bacteria were identified abundantly in digestive tracts of animals, several species of this family helps in the production of butyric acid, which is important for both microbial and host epithelial cell growth [20]. *Eryspelotrichaceae* was the second most abundant family (19%) in the chicken. Family *Erysipelotrichaceae* also belongs to the phylum *Firmicutes* and was identified from the gut microbiome. Research shows that these family members are associated with obesity [21], which is also a significant problem and liability in the broiler production industry and health conscious consumers [22]. *Eryspelotrichaceae* family was followed by *Coriobacteriaceae* (16%), *Lactobacillaceae* (13%), *Ruminococaceae* (7%) and *Clostridiaceae* (7%).

In contrast to chickens, Guinea fowls had 20 families of bacteria that constituted 1% or more of the gut flora, accounting for 94% of the flora. *Prevotellaceae* was the most abundant (12%), followed by *Enterobacteriaceae* (11%) and *Bacteroidaceae* (11%). Apparently, the Simpson index for Guinea fowls was higher than that for chickens. *Prevotellaceae* belongs to phylum *Bacteroidetes*, a family of bacteria known to aid the breakdown of protein and carbohydrate foods and usually found in the gut of animals. Low levels of *Prevotellaceae* group members were identified in patients with Parkinson’s disease (PD); research is still ongoing to establish the relation between members of the *Prevotellaceae* family and the patients of PD. Further in-depth research is required to understand the beneficial effects of *Prevotellaceae* family [23]. *Enterobacteriaceae* is a large family of gram negative bacteria which belongs to the phylum *Proteobacteria*. Species of *Enterobacteriaceae* are regularly found in intestines of animals, mostly harmless, while some of them are symbiotic and some others including *Salmonella*, *Escherichia coli* etc. are pathogenic.

**Fig 3:**
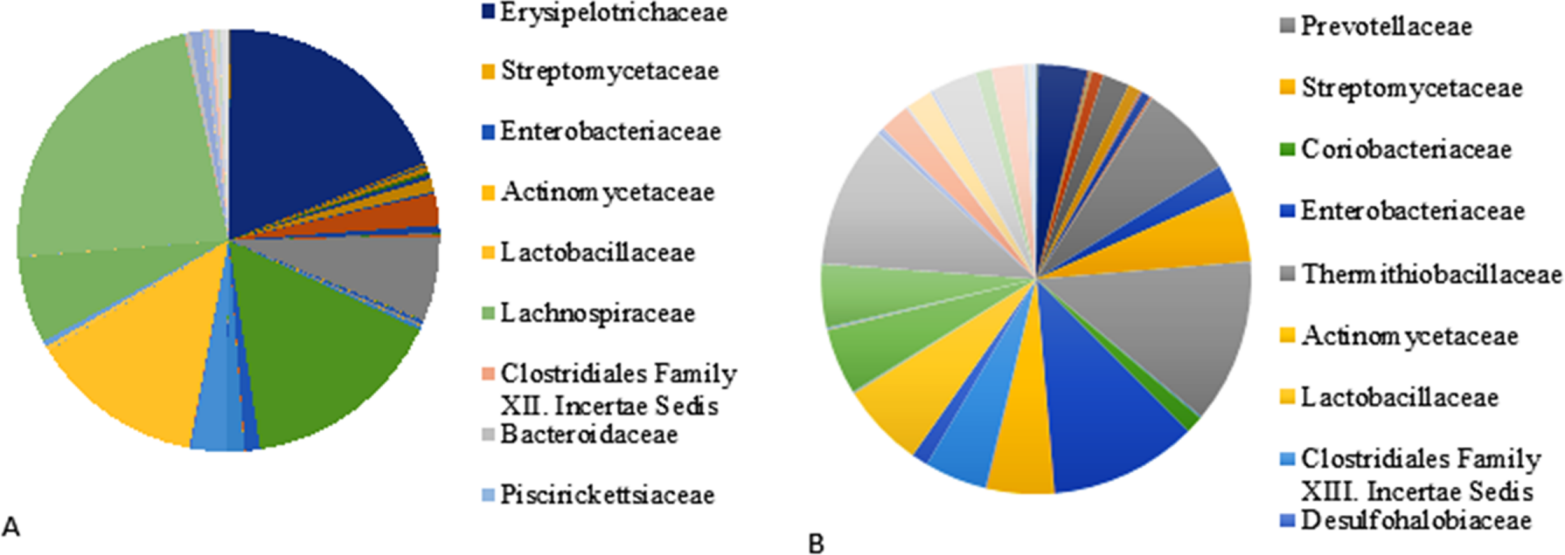
Phylogenetic family distribution of microbial profile derived from sequencing the 16s rRNA gene of a metagenomics library of chickens (A) and Guinea fowl (B) GIT contents. The percent of reads in each of the bacterial family from the GIT microbiome is shown. E-value cutoff for 16s rRNA hits for all databases used is 1x10^-5^ with a minimum length of 50 bp. The BLASTX cutoff for gene tags is 1x 10^−5^.

The most abundant chicken microflora families were also relatively abundant in the Guinea fowl, but the most abundant Guinea fowl microbes were much less abundant in the chickens (Fig 3 and S1 Table). The family *Areococcaceae* contributed 1.32% to the chicken flora, but was not found in Guinea fowls. On the other hand, *Sutterellaceae* constituted 2.44% of the Guinea fowl gut flora, but was not found in chickens. Overall, there were 36 unique Guinea fowl flora families not in the chicken gut flora, and 35 unique chicken microbial families not found in the Guinea fowl gut flora. None of the other single host bacterial families contributed more than 1% to the gut flora of either chickens or Guinea fowls.

#### 2.2 Species profile

Although only a small portion of all the mapped reads could be identified at the species level, it is of interest to compare the species identified in the two host species. In total, seventy nine (79) species were identified from the chicken libraries and 53 from the Guinea fowl libraries. Among the species in chickens, 43 species were not found in the Guinea fowl. Conversely, there were only 15 species in the Guinea fowls that were not found in chickens. We calculated binomial probability distribution for a given species at 100 cells per million bacteria. The probability of seeing no more than 10 cells (bioinformatics cutoff) of the given species in one million trials was smaller than 10^−6^. Thus, we considered it being statistically significant if any bacterial species, with a frequency of 0.01% (100 per million) in one host, was not found in the other host. With this conservative estimate, we determined that 23 chicken bacterial species, out of 43, were proliferated in the chicken gut, but not in the Guinea fowls. Conversely, 8 Guinea fowl bacterial species proliferated in the Guinea fowl gut, but not in chickens (S1 Table).

The most abundant single bacterial species was *Clostridium spiroforme* [24] of *Erysipelotrichaceae* family (phylum *Firmicutes*), which contributed 7.9% of all mapped reads in chickens. These bacterial species were abundantly identified in the chicken gut and were not found in Guinea fowls. Some strains of *C*. *spiroforme* have been reported to produce endotoxins and cause diarrhea in rabbits [25, 26]. However, the strain in the chickens may be harmless because the experimental birds appeared healthy.

*Eubacterium xylanophilum* was found in the Guinea fowl gut in considerable abundance (0.1% of total mapped reads), but not in chickens. This distribution suggested *that E*. *xylanophilum* colonized in Guinea fowls, but not in chickens, despite the fact that these chickens were raised in close contact with the Guinea fowls. This bacterium degrades xylem, but not cellulose [27]. It is often found in the rumen of ruminants and the digestive tract of other mammals including humans. In contrast, *Eubacterium cylindroides* [28] was found to be abundant in chickens (1.4% of total mapped reads), but less abundant in the Guinea fowls.

*Parasutteralla secunda* is a recently identified species; it has been isolated from human feces [29]. This species was abundant in the Guinea fowls (0.6%), but not found in the chickens. To our knowledge, this is the first report of *P*. *Secunda* in aves.

*Clostridium perfringens* was found in both chickens and Guinea fowls at low frequencies. This pathogenic bacterium is considered a commensal species of the intestine, but proliferation of type A of *C*. *perfringens* and release of toxin result in necrotic enteritis in poultry [30, 31]. *Salmonella enterica* and *Escherichia coli* was found in the Guinea fowls at very low frequency, not in the chickens. It is not surprising to find these common species in Guinea fowls in view of previous reports [32, 33].

#### 2.3 Lactobacillaceae

**Fig 4:**
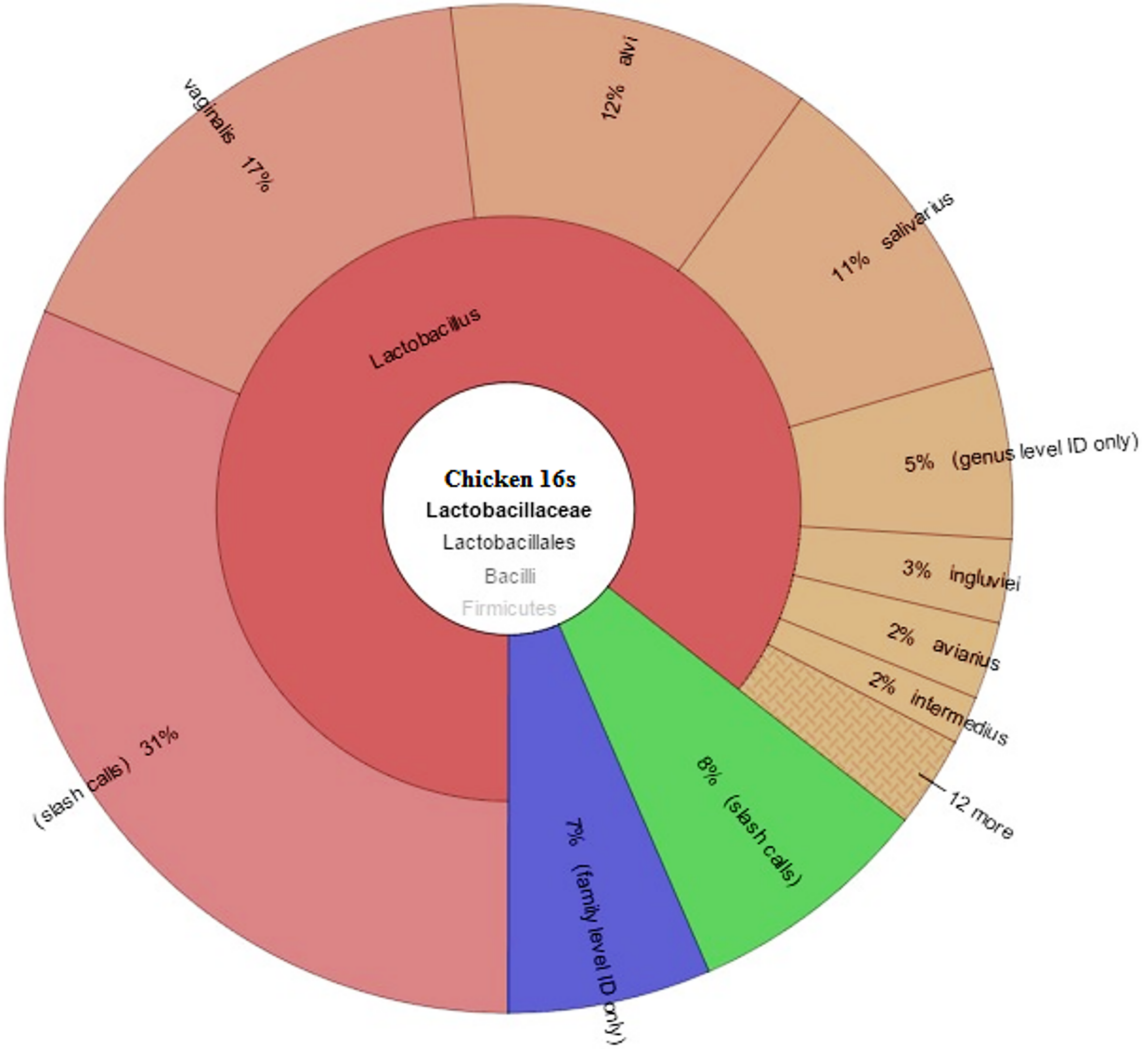
Chicken *Lactobacillaceae* family distribution of microbial profile derived from sequencing the 16s rRNA gene of a metagenomics library of chicken GIT contents.

*Lactobacillaceae* family members are highly accepted as probiotics which help to maintain the gut health of birds. The lactobacilli colonizing the intestine of birds may secrete enzymes such as amylase, thus increasing the intestinal amylase activity [34]. It is well established that these bacteria alter gastrointestinal pH and flora to favor an increased activity of intestinal enzymes and digestibility of nutrients [35]. In this study *Lactobacillaceae* family is identified in both microbial profiles of chicken and Guinea fowl. The abundance of this family is different in both avian species. In the chicken gut profile, *Lactobacillaceae* family constituted 12% or 286,737 reads of the total 2,389,475 reads generated from the chicken gut profile. The family belongs to the phylum *Firmicutes* and the class *Bacilli* (Fig 4). This family group is mostly abundant in the digestive tract of animals and produce lactic acid as an end product in carbohydrate metabolism. Thus these bacteria are also known to be acid tolerant and they help to maintain gut health. Most species of this family are well known and widely utilized as probiotics. In chicken profile maximum species level identification was observed in the family of *Lactobacillaceae* (Table 3). Eighteen species identified in the gut of chicken belong to *Lactobacillaceae* family with the identification range of 99.1–100%. *Lactobacillus vaginallis* was most abundant with 38,091 reads and represented 20% of the family, followed by *L*. *alvi* (14%) and *L*. *salivarius* (12%). *L*. *vaginallis* comes from the complex of *L*.*acidophillus*, which are known to protect the host from urogenital infections [36].

**Table 3:** *Lactobacillaceae* species level identification in the chicken gut.

Genus	Species	ID %	Count	% count
Lactobacillus	agilis	99.2–100	1569	0.6
Lactobacillus	alvi	99.12–100	25882	14
Lactobacillus	aviaries	99.12–100	4154	3
Lactobacillus	coleohominis	99.19–100	412	0.2
Lactobacillus	crispatus	100–100	41	0.01
Lactobacillus	delbrueckii	99.17–100	1337	0.9
Lactobacillus	Equi	99.1–99.1	117	0.1
Lactobacillus	ingluviei	99.12–100	7068	3
Lactobacillus	intermedius	99.1–100	2634	2
Lactobacillus	mucosae	99.17–100	1390	0.6
Lactobacillus	oris	100–100	60	0.03
Lactobacillus	panis	99.13–99.13	196	0.3
Lactobacillus	pontis	99.16–100	637	0.3
Lactobacillus	reuteri	99.1–100	692	0.3
Lactobacillus	saerimneri	99.1–100	587	0.3
Lactobacillus	salivarius	99.1–100	23293	12
Lactobacillus	secaliphilus	99.12–100	145	0.06
Lactobacillus	sp.	100–100	11	0.01
Lactobacillus	vaginalis	99.12–100	38091	20

**Fig 5:**
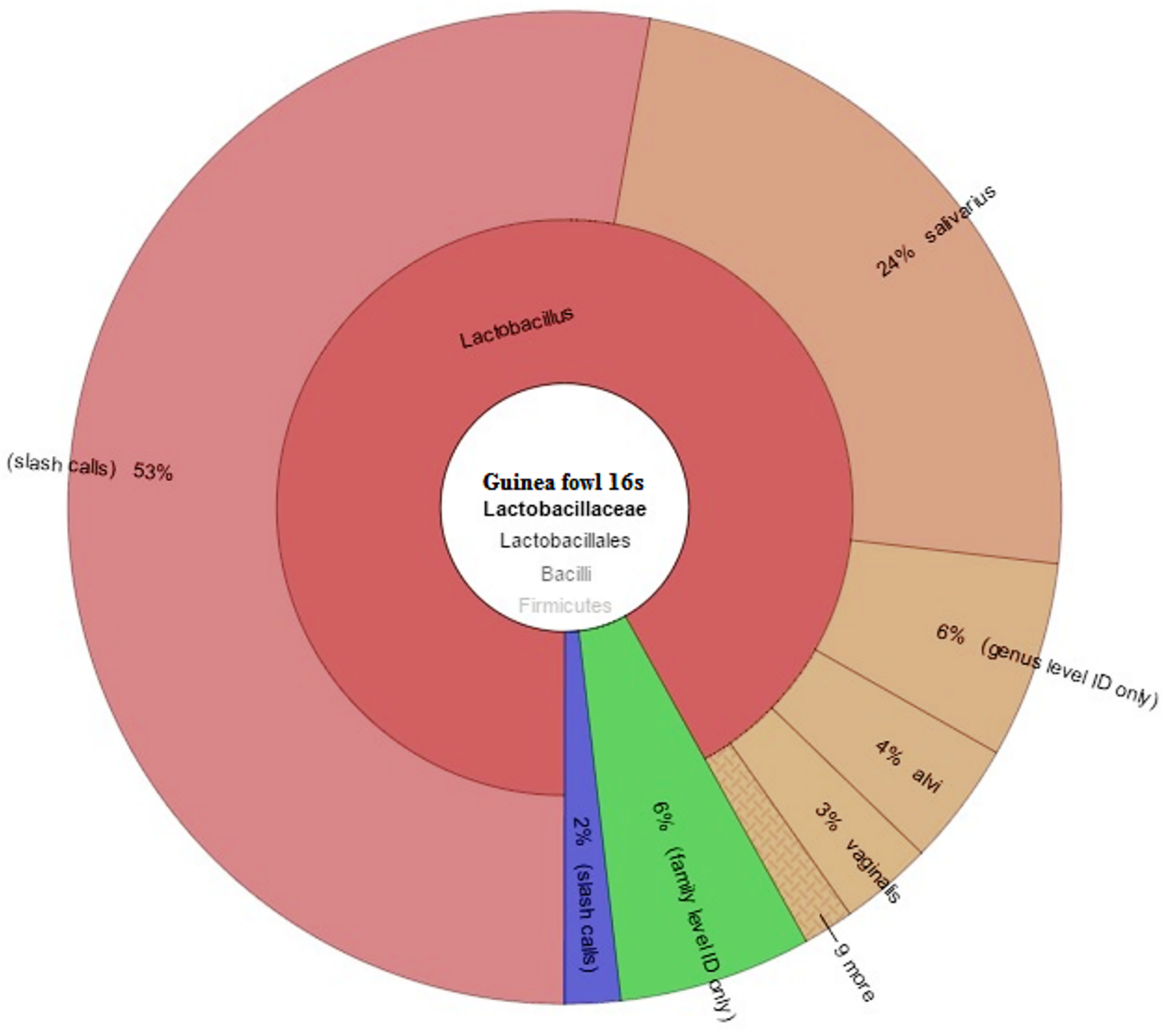
Guinea fowl *Lactobacillaceae* family distribution of microbial profile derived from sequencing the 16s rRNA gene of a metagenomics library of guinea fowl GIT contents.

In the gut of Guinea fowl, the *Lactobacillaceae* family constituted about 6% of the total flora or 65,645 reads of the total 1,094,083 reads (Fig 5). Similar to reads in chickens, some reads of the *Lactobacillaceae* family in Guinea fowl were also identified up to species level. A total of 11 species of the *Lactobacillaceae* family were identified in the profile of Guinea fowl (Table 4). *L*. *salivarius* was identified with maximum of 9,866 reads, constituting about 26% of the total reads from the family, followed by *L*. *alvi* (4%) and *L*. *vaginalis* (3%). *L*. *salivarius* is a well-established gastrointestinal tract probiotic bacterium. Research indicates that this bacterium has a beneficial effect on treating the irritable bowel syndrome and pancreatic necrosis. There is still significant ongoing research to understand the antimicrobial properties of *L*. *salivarius*. Many of these species were found in high abundance, each constituting 0.1% or more of the gut microbiota.

**Table 4:** *Lactobacillaceae* species level identification in the Guinea fowl gut.

Genus	Species	% ID	Count	% count
Lactobacillus	agilis	99.2–100	252	0.4
Lactobacillus	alvi	99.12–100	1985	4
Lactobacillus	aviarius	99.6–100	30	0.07
Lactobacillus	crispatus	100–100	15	0.01
Lactobacillus	equi	99.1–99.11	44	0.05
Lactobacillus	frumenti	99.6–100	92	0.1
Lactobacillus	ingluviei	99.19–100	459	0.7
Lactobacillus	pontis	99.6–100	24	0.04
Lactobacillus	reuteri	99.11–99.58	168	0.3
Lactobacillus	salivarius	99.1–100	9866	26
Lactobacillus	vaginalis	99.17–100	1942	3

#### 2.4 Bifidobacteriaceae

**Fig 6:**
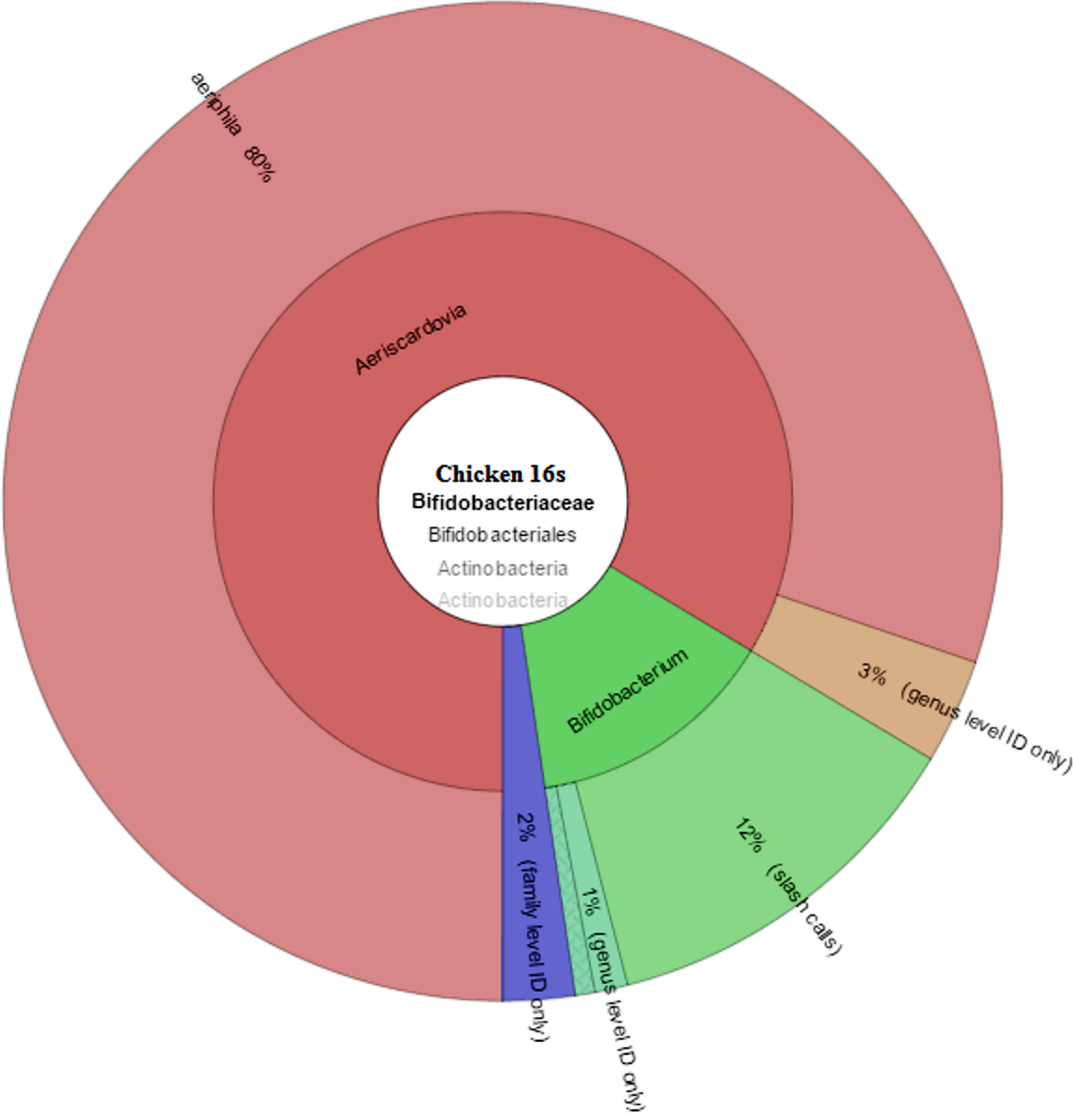
Chicken *Bifidobacteriaceae* family distribution of microbial profile derived from sequencing the 16s rRNA gene of a metagenomics library of chicken GIT contents.

**Fig 7:**
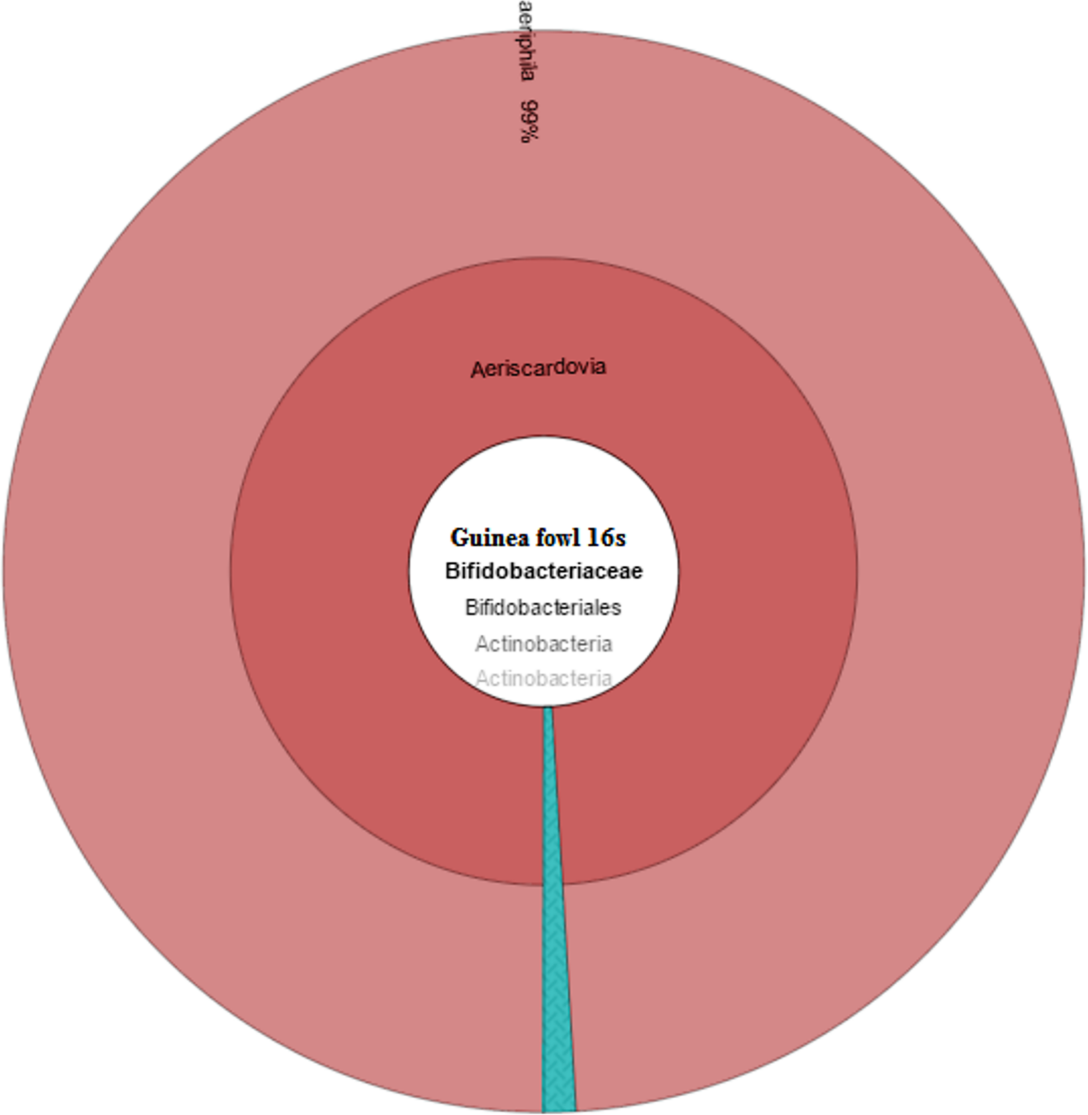
Guinea fowl *Bifidobacteriaceae* family distribution of microbial profile derived from sequencing the 16s rRNA gene of a metagenomics library of guinea fowl GIT contents.

The *Bifidobacteriaceae* family belongs to phylum *Actinobacteria* and order *Bifidobacteriales*. Members of this family are naturally found in the guts of animals and humans, and are regarded as beneficial to the human health and widely used as probiotic bacteria [37]. In chicken gut profile, the *Bifidobacteriaceae* family consisted of 0.5% with 11,480 reads. Only 3 species were identified in chickens for this family (Table 5 and Fig 6). In this family *Aeriscardovia aeriphila* was highly abundant and accounted for 80% of the family. This bacterium was isolated and identified in porcine caecum [38] but still not much information is available regarding this bacterium. In Guinea fowl *Bifidobacteriaceae* family consists of 0.1% of the gut profile. Only *Aeriscardovia aeriphila* was identified in Guinea fowl profile and it consisted of 99% of *Bifidobacteriaceae* family (Table 6 and Fig 7).

**Table 5:** *Bifidobacteriaceae* species level identification in the chicken gut.

Family	Genus	Species	%ID	Count	% Count
Bifidobacteriaceae	Aeriscardovia	Aeriphila	99–100	8654	80
Bifidobacteriaceae	Bifidobacterium	(genus only)		254	4
Bifidobacteriaceae	Bifidobacterium	(slash calls)		1293	12
Bifidobacteriaceae	Bifidobacterium	pseudolongum	100–100	37	2
Bifidobacteriaceae	Bifidobacterium	pullorum	99–99	38	2

**Table 6:** *Bifidobacteriaceae* species level identification in Guinea fowl gut.

Family	Genus	Species	%ID	Count	% Count
Bifidobacteriaceae	Aeriscardovia	(genus)		349	
Bifidobacteriaceae	Aeriscardovia	aeriphila	99–100	517	99
Bifidobacteriaceae	Bifidobacterium	(slash)		11	

## Conclusion

The gut microbiota is influenced by many factors, including host species immunity, developmental stage, diets and history of contact with environmental microbes. In this study we compared the gut microbiota of age matched chicken and guinea fowls with no history of disease housed in similar condition and fed identical diets. Thus, the specific bacterial profile differences reported here have largely resulted from the host and microbe interactions. This study provides a basic reference for the gut microbiome of both chickens and Guinea fowls and provides the first comprehensive study of the microbiota in the gastrointestinal tract of Guinea fowls. Our findings demonstrate that the presence of *Verrucomicrobia* and *Lentisphaerae* in the guinea fowl GIT is a point of differentiation between the two species. This study supports the research on species specific gut microbes and also provides information to evaluate the applications of probiotic microbes in the gut of chickens and Guinea fowls.

## Supporting information

S1 Table Taxonomy of identified bacterial families in chicken and Guinea fowl gut profiles. (PDF)

S1 File Chicken 16SrRNA Results 1. 16SrRNA sequencing data revealing Intestinal microbial profile of the chicken gastrointestinal tract.
(ZIP)

S2 File Chicken 16SrRNA Results 2. 16SrRNA sequencing data revealing Intestinal microbial profile of the chicken gastrointestinal tract.
(ZIP)

S3 File Guinea fowl 16SrRNA Results 1. 16SrRNA sequencing data revealing Intestinal microbial profile of the guinea fowl gastrointestinal tract.
(ZIP)

S4 File Guinea fowl 16SrRNA Results 2. 16SrRNA sequencing data revealing Intestinal microbial profile of the guinea fowl gastrointestinal tract.
(ZIP)
