# Supplementary material for: A comparative analysis of microbial profile of Guinea fowl and chicken using metagenomic approach
Source: PLoS One. 2018 Mar 1;13(3):e0191029. doi: 10.1371/journal.pone.0191029 (PMC5832216; doi:10.1371/journal.pone.0191029)
Supplement: S1 Table — (PDF) [file pone.0191029.s001.pdf]

Supplement Table:

Taxonomy of identified bacterial families in chicken and Guinea fowl gut profiles

**FAMILY**	**Chick 16S reads**	**GF 16S reads**	**Chick reads**	**GF reads**
**Phylum: Proteobacteria**	-------------------(count)--------------------		-------------------------(%)---------------------	
**Class: Alphaproteobacteria**				
**Order: Rhizobiales**				
Xanthobacteraceae	0	31.00	0 ^{b}	29.67^{a}
Rhizobiaceae	0	23.00	0 ^{b}	22.02^{a}
Methylobacteriaceae	17.00	60.00	7.59^{b}	57.44^{a}
Hyphomicrobiaceae	7789.00	441.00	3475.50^{b}	422.15^{b}
Bartonellaceae	0	18.00	0^{b}	17.23^{a}
Beijerinckiaceae	25.00	11.00	11.16^{a}	10.53^{a}
Cohaesibacteraceae	0	12.00	0 ^{b}	11.49^{a}
**Order: Caulobacterales**				
Caulobacteraceae	0	74.00	0^{b}	70.84^{a}
**Order: Rhodospirillales**				
Acetobacteraceae	0	423.00	0 ^{b}	404.92^{a}
**Order: Sphingomonadales**				
Sphingomonadaceae	19.00	0	8.48^{a}	0^{b}
**Class: Gammaproteobacteria**				
**Order: Xanthomonadales**				
Xanthomonadaceae	0	1037.00	0^{b}	992.67^{a}
**Order: Vibrionales**				
Vibrionaceae	0	159.00	0^{b}	152.20^{a}
**Order: Thiotrichales**				
Thiotrichaceae	165.00	56.00	73.62^{a}	53.61^{b}
**Order: Aeromonadales**				
Succinivibrionaceae	146.00	23868.00	65.15^{b}	22847.73^{a}
**Order: Alteromonadales**				
Shewanellaceae	0	20.00	0^{b}	19.15^{a}
Thermithiobacillaceae	0	84.00	0^{b}	80.41^{a}
**Order: Thiotrichales**				
Piscirickettsiaceae	45.00	954.00	20.08^{b}	913.22^{a}
**Order: Pasteurellales**				
Pasteurellaceae	62.00	9126.00	27.66^{b}	8735.90a
**Order: Oceanospirillales**				
Oceanospirillaceae	0	13.00	0^{b}	12.44^{a}
Oleiphilaceae	0	18.00	0 ^{b}	17.23^{a}
**Order: Pseudomonadales**				

Moraxellaceae	373.00	14.00	166.43[a]	13.40[b]
**Order: Methylococcales**				
Methylococcaceae	28.00	49.00	12.49[b]	46.91[a]
**Order: Legionellales**				
Legionellaceae	97.00		43.28[a]	0[b]
**Order: Oceanospirillales**				
Halomonadaceae	13.00	42.00	5.80[b]	40.20[a]
**Order: Chromatiales**				
Ectothiorhodospiraceae	0	10.00	0[b]	9.57[a]
**Order: Cardiobacteriales**				
Cardiobacteriaceae	0	176.00	0[b]	168.48[a]
**Order:Oceanospirillales**				
Alcanivoracaceae	23.00	0	10.26[a]	0 [b]
**Order: Aeromonadales**				
Aeromonadaceae	330.00	0	147.25[a]	0 [b]
**Order: Rhodobacterales**				
Rhodobacteraceae	1428.00	100.00	637.18[a]	95.73[b]
**Order: Rhodospirillales**				
Rhodospirillaceae	144.00	448.00	64.25[b]	428.85[a]
**Order: Holosporales**				
Holosporaceae	26.00	240.00	11.60[b]	229.74[a]
**Order: Sphingomonadales**				
Erythrobacteraceae	462.00	0	206.15[a]	0 [b]
**Order: Enterobacteriales**				
Enterobacteriaceae	25983.00	116181.00	11,593.77[b]	111,214.71[a]
**Class: Deltaproteobacteria**				
**Order: Syntrophobacterales**				
Syntrophobacteraceae	10.00	0	4.46[a]	0[a]
**Order: Desulfuromonadales**				
Pelobacteraceae	122.00	0	54.44[a]	0[b]
Geobacteraceae	2917.00	115.00	1301.58[a]	110.08[b]
**Order: Pseudomonadales**				
Pseudomonadaceae	397.00	11.00	177.14[a]	10.53[b]
**Order: Desulfovibrionales**				
Desulfohalobiaceae	0	19.00	0[b]	18.19[a]
Desulfovibrionaceae	6382.00	35043.00	2,847.69[b]	33,545.05[a]
**Order: Desulfuromonadales**				
Desulfuromonadaceae	11.00	0	4.91[a]	0[a]
**Class: Betaproteobacteria**				
**Order: Rhodocyclales**				

Rhodocyclaceae	27.00	715.00	12.05[b]	684.44[a]
**Order: Burkholderiales**				
Oxalobacteraceae	550.00	21767.00	245.41[b]	20,836.54[a]
Burkholderiaceae	0	340.00	0 [b]	325.47[a]
Alcaligenaceae	1102.00	675.00	491.72[b]	646.15[a]
Comamonadaceae	0	392.00	0[b]	375.24[a]
Sutterellaceae	0	25524.00	0 [b]	24,432.95[a]
**Order: Neisseriales**				
Neisseriaceae	0	117.00	0 [b]	112.00[a]
**Order: Nitrosomonadales**				
Spirillaceae	0	13.00	0[b]	12.44[a]
**Class: Epsilonproteobacteria**				
**Order: Campylobacterales**				
Helicobacteraceae	667.00	304.00	297.62[a]	291.01[a]
unclassified Campylobacterales	20.00	0	8.92[a]	0[b]
**Phylum:Bacteroidetes**				
**Class: Bacteroidia**				
**order: Bacteroidales**				
[Paraprevotellaceae]	143.00	1095.00	63.81[b]	1048.19[a]
**Class: Bacteroidetes**				
Bacteroidaceae	9,899.00	111,077.00	4,416.99[b]	10,6328.88[a]
Marinilabiliaceae	0	12.00	0 [b]	11.49[a]
Porphyromonadaceae	959.00	56204.00	427.91[b]	53,801.49[a]
Prevotellaceae	4300.00	129233.00	1,918.69[b]	123,708.78[a]
Prolixibacteraceae	0	10.00	0 [b]	9.57[a]
Rikenellaceae	0	2248.00	0 [b]	2151.91[a]
**Class: Sphingobacteriia**				
**Order: Sphingobacteriales**				
Flammeovirgaceae	0	77.00	0 [b]	73.71[a]
Saprospiraceae	15.00	3330.00	6.69[b]	3187.66[a]
Sphingobacteriaceae	0	134.00	0[b]	128.27[a]
Chitinophagaceae	0	10.00	0[b]	9.57[a]
**Class: Flavobacteriia**				
**Order: Flavobacteriales**				
Cryomorphaceae	19.00	347.00	8.48[b]	332.17[a]
**Class: Cytophagia**				
**Order: Cytophagales**				
Cytophagaceae	32.00	2266.00	14.28[b]	2169.14[a]
**Phylum: Firmicutes**				
**Class: Mollicutes**				

**Order: Acholeplasmatales**				
Acholeplasmataceae	636.00	199.00	283.79[a]	190.49[b]
**Order: Anaeroplasmatales**				
Anaeroplasmataceae	0	13.00	0[b]	12.44[a]
**Class: Negativicutes**				
**Order: Selenomonadales**				
Acidaminococcaceae	825.00	21280.00	368.12[b]	20,370.36[a]
Veillonellaceae	1887.00	13088.00	841.99[b]	12,528.54[a]
**Class: Bacilli**				
**Order: Lactobacillales**				
Aerococcaceae	29630.00	0	13,221.08[a]	0[b]
Carnobacteriaceae	7272.00	62.00	3,244.81[a]	59.35[b]
Enterococcaceae	53623.00	1490.00	23,926.90[a]	1,426.31[b]
Leuconostocaceae	505.00	33.00	225.33[a]	31.59[b]
Lactobacillaceae	296806.00	65700.00	132,436.58[a]	62,891.58[b]
Streptococcaceae	12630.00	6956.00	5,635.58[b]	6,658.66[a]
**Order: Bacillales**				
Alicyclobacillaceae	16.00	22.00	7.14[b]	21.06[a]
Bacillaceae	10011.00	932.00	4,466.97[a]	892.16[b]
Bacillales incertae sedis	565.00	11.00	252.11[a]	10.53[b]
Listeriaceae	37.00	0	16.51[a]	0 [b]
Paenibacillaceae	8741.00	817.00	3,900.29[a]	782.08[b]
Planococcaceae	2081.00	259.00	928.55[a]	247.93[b]
Staphylococcaceae	10248.00	931.00	4,572.72[a]	891.20[b]
unclassified Clostridiales	509.00	1427.00	227.12[b]	1,366.00[a]
Thermoactinomycetaceae	1021.00	24.00	455.58[a]	22.97[b]
**Class: Clostridia**				
**Order : Clostridiales**				
Caldicoprobacteraceae	43.00	0	19.19[a]	0[b]
Catabacteriaceae	2523.00	449.00	1,125.78[a]	429.81[b]
Christensenellaceae	5133.00	554.00	2,290.37[a]	530.32[b]
Clostridiaceae	145418.00	74407.00	64,886.36[b]	71,226.39[a]
Clostridiales Family XI. Incertae Sedis	0	10.00	0[b]	9.57[a]
Clostridiales Family XII. Incertae Sedis	194.00	0	86.56[a]	0[b]
Clostridiales Family XIII. Incertae Sedis	414.00	0	184.73[a]	0[b]
Clostridiales Family XVI. Incertae Sedis	155.00	137.00	69.16[b]	131.14[a]
Defluviitaleaceae	117.00	0	52.21[a]	0[b]
Eubacteriaceae	60585.00	49064.00	27,033.38[b]	46,966.70[a]
Gracilibacteraceae	470.00	78.00	209.72[a]	74.67[b]
Heliobacteriaceae	10.00	0	4.46[a]	0[a]

Lachnospiraceae	511662.00	49795.00	228,306.59[a]	47,666.45[b]
Oscillospiraceae	147.00	3491.00	65.59[b]	3,341.77[a]
Peptococcaceae	19383.00	2732.00	8,648.81[a]	2,615.22[b]
Peptostreptococcaceae	21694.00	9624.00	9,679.99[a]	9,212.61[b]
Proteinivoraceae	12.00	348.00	5.35[b]	333.12[a]
Ruminococcaceae	149411.00	51650.00	66,668.06[a]	49,442.16[b]
Syntrophomonadaceae	360.00	168.00	160.63[a]	160.82[a]
**Unclassified Bacteria**				
Caldithrix	24.00	0	10.71[a]	0[b]
**Class: Erysipelotrichia**				
**Order: Erysiopelotrichales**				
Erysipelotrichaceae	422304.00	38676.00	188,434.52[a]	37,022.75[b]
**Order: Halanaerobiales**				
Halanaerobiaceae	105.00	0	46.85[a]	0[b]
Halobacteroidaceae	704.00	66.00	314.13[a]	63.18[b]
**Order: Thermoanaerobacterales**				
Thermoanaerobacteraceae	53.00	41.00	23.65[b]	39.25[a]
Thermoanaerobacterales Family III. Incertae Sedis	29.00	0	12.94[a]	0[b]
Thermoanaerobacterales Family IV. Incertae Sedis	0	52.00	0[b]	49.78[a]
**Class: Thermolithobacteria**				
**Order: Thermolithobacterales**				
Thermolithobacteraceae	0.00	10.00	0.00[a]	4.46[a]
**Class: Flavobacteria**				
**Order: Flavobacteriales**				
Flavobacteriaceae	301.00	51838.00	134.31[b]	49,622.12[a]
**Phylum: Actinobacteria**				
**Class: Actinobacteria**				
**Order: Acidimicrobiales**				
Acidimicrobiaceae	29.00	0	12.94[a]	0[b]
**Order: Actinomycetales**				
Actinomycetaceae	16.00	0	7.14[a]	0[b]
Brevibacteriaceae	502.00	0	224.00[a]	0[b]
Dermabacteraceae	44.00	0	19.63[a]	0[b]
Dermacoccaceae	0	46.00	0[a]	44.03[b]
Dietziaceae	219.00	0	97.72[a]	0 [b]
Geodermatophilaceae	16.00	87.00	7.14[b]	83.28[a]
Intrasporangiaceae	4315.00	0	1,925.38[a]	0[b]
Kineosporiaceae	52.00	0	23.20[a]	0[b]
Micrococcaceae	484.00	0	215.96[a]	0[b]
Micromonosporaceae	0	11.00	0[b]	10.53[a]

Microthrixaceae	84.00	0	37.48[a]	0[b]
Nakamurellaceae	284.00	0	126.72[a]	0[b]
Nocardioidaceae	46.00	15.00	20.53[a]	14.36[a]
Patulibacteraceae	0	10.00	0[b]	9.57[a]
Propionibacteriaceae	13.00	0	5.80[a]	0[a]
Pseudonocardiaceae	17.00	0	7.59[a]	0[b]
Streptomycetaceae	838.00	81.00	373.92[a]	77.54[b]
Thermomonosporaceae	27.00	0	12.05[a]	0[b]
**Order: Bifidobacteriales**				
Bifidobacteriaceae	12190.00	1091.00	5,439.25[a]	1,044.36[b]
**Order: Coriobacteriales**				
Coriobacteriaceae	350317.00	13938.00	156,313.50[a]	13,342.20[b]
**Order: Corynebacteriales**				
Corynebacteriaceae	6453.00	12.00	2,879.37[a]	11.49b
**Phylum: Spirochaetes**				
**Class: Spirochaetia**				
**Order: Spirochaetales**				
Brachyspiraceae	3404.00	1609.00	1,518.88[a]	1,540.22[a]
Spirochaetaceae	120.00	20695.00	53.54[b]	19,810.37[a]
**Phylum: Deferribacteres**				
**Class: Deferribacteres**				
**Order: Deferribacterales**				
Deferribacteraceae	75.00	114.00	33.47[b]	109.13[a]
**Phylum: Chloroflexi**				
**Class: Dehalococcoidetes**				
**Order: Dehalococcoidales**				
Dehalococcoidaceae	5506.00	1177.00	2,456.81[a]	1,126.69[b]
**Class: Ktedonobacteria**				
**Order: Ktedonobacterales**				
Thermosporotrichaceae	165.00	21.00	73.62[a]	20.10[b]
**Class: Dehalococcoidia**				
unclassified Dehalococcoidia	1402.00	365.00	625.58[a]	349.40[b]
**Phylum: Tenericutes**				
**Class: Mollicutes**				
**Order: Entomoplasmatales**				
Spiroplasmataceae	551.00	33.00	245.86a	31.59[b]
Entomoplasmataceae	2616.00	0	1,167.27[a]	0b
**Order: Haloplasmatales**				
Haloplasmataceae	291.00	0	129.85[a]	0[b]
**Order: Mycoplasmatales**				

Mycoplasmataceae	3198.00	28.00	1,426.97^{a}	26.80^{b}
**Phylum: Fusobacteria**				
**Class: Fusobacteria**				
**Order: Fusobacteriales**				
Leptotrichiaceae	55.00	0	24.54^{a}	0^{b}
**Phylum: Cyanobacteria**				
**Class: Cyanophyceae**				
**Order: Nostocales**				
Nostocaceae	1493.00	442.00	666.19a	423.11^{b}
**Order: Oscillatoriales**				
Phormidiaceae	24.00	124.00	10.71^{b}	118.70a
**Phylum: Verrucomicrobia**				
**Class: Opitutae**				
**Order: Opitutales**				
Opitutaceae	0	279.00	0^{b}	267.07^{a}
**Order: Puniceicoccales**				
Puniceicoccaceae	0	584.00	0^{b}	559.04^{a}
**Class: Verrucomicrobiae**				
**Order: Verrucomicrobiales**				
Verrucomicrobia subdivision 3	0	11.00	0^{b}	10.53^{a}
**Phylum: Synergistetes**				
**Class: Synergistia**				
**Order: Synergistales**				
Synergistaceae	3179.00	12612.00	1,418.49^{a}	1,2072.89^{b}
**Phylum: Thermotogae**				
**Class: Thermotogae**				
**Order: Thermotogales**				
Thermotogaceae	52.00	0	23.20^{a}	0^{b}
**Phylum: Lentisphaerae**				
**Class: Lentisphaeria**				
**Order: Victivallales**				
Victivallaceae	0	70.00	0^{b}	67.01^{a}

GF-Guinea fowl.

Chik- Chicken.

^{a,b} Mean percent reads within rows with no common superscripts differ significantly (P<0.05).
