# Supplementary material for: A comparative analysis of microbial profile of Guinea fowl and chicken using metagenomic approach
Source: PLoS One. 2018 Mar 1;13(3):e0191029. doi: 10.1371/journal.pone.0191029 (PMC5832216; doi:10.1371/journal.pone.0191029)
Supplement: S1 File — 16SrRNA sequencing data revealing Intestinal microbial profile of the chicken gastrointestinal tract. (ZIP) [file pone.0191029.s002.zip › exports/alphaDiversityDir_species/alpha_rarefaction_plots/rarefaction_plots.html]

Rarefaction Curves


|  |  |  |  |
| --- | --- | --- | --- |
| **Select a Metric:** | chao1 observed\_species shannon simpson | **Select a Category:** | SampleID |

  

**Show Categories:
 
All
None
Invert**

**Legend**

|  |  |  |  |
| --- | --- | --- | --- |
| ▶ |  | ■ | **S001\_chic\_2\_sample\_16s\_10-23-15\_v2** |
| ∟ |  | ◆ | **S001\_chic\_2\_sample\_16s\_10-23-15\_v2** |
| ▶ |  | ■ | **S002\_Sarayu\_Chic\_16s\_2-Sample\_10-30-15\_v1** |
| ∟ |  | ◆ | **S002\_Sarayu\_Chic\_16s\_2-Sample\_10-30-15\_v1** |
| ▶ |  | ■ | **S003\_GF\_2\_SAMPLE\_v1** |
| ∟ |  | ◆ | **S003\_GF\_2\_SAMPLE\_v1** |
| ▶ |  | ■ | **S004\_GF\_16S\_2\_v1** |
| ∟ |  | ◆ | **S004\_GF\_16S\_2\_v1** |
| ▶ |  | ■ | **S001\_chic\_2\_sample\_16s\_10-23-15\_v2** |
| ∟ |  | ◆ | **S001\_chic\_2\_sample\_16s\_10-23-15\_v2** |
| ▶ |  | ■ | **S002\_Sarayu\_Chic\_16s\_2-Sample\_10-30-15\_v1** |
| ∟ |  | ◆ | **S002\_Sarayu\_Chic\_16s\_2-Sample\_10-30-15\_v1** |
| ▶ |  | ■ | **S003\_GF\_2\_SAMPLE\_v1** |
| ∟ |  | ◆ | **S003\_GF\_2\_SAMPLE\_v1** |
| ▶ |  | ■ | **S004\_GF\_16S\_2\_v1** |
| ∟ |  | ◆ | **S004\_GF\_16S\_2\_v1** |
| ▶ |  | ■ | **S001\_chic\_2\_sample\_16s\_10-23-15\_v2** |
| ∟ |  | ◆ | **S001\_chic\_2\_sample\_16s\_10-23-15\_v2** |
| ▶ |  | ■ | **S002\_Sarayu\_Chic\_16s\_2-Sample\_10-30-15\_v1** |
| ∟ |  | ◆ | **S002\_Sarayu\_Chic\_16s\_2-Sample\_10-30-15\_v1** |
| ▶ |  | ■ | **S003\_GF\_2\_SAMPLE\_v1** |
| ∟ |  | ◆ | **S003\_GF\_2\_SAMPLE\_v1** |
| ▶ |  | ■ | **S004\_GF\_16S\_2\_v1** |
| ∟ |  | ◆ | **S004\_GF\_16S\_2\_v1** |
| ▶ |  | ■ | **S001\_chic\_2\_sample\_16s\_10-23-15\_v2** |
| ∟ |  | ◆ | **S001\_chic\_2\_sample\_16s\_10-23-15\_v2** |
| ▶ |  | ■ | **S002\_Sarayu\_Chic\_16s\_2-Sample\_10-30-15\_v1** |
| ∟ |  | ◆ | **S002\_Sarayu\_Chic\_16s\_2-Sample\_10-30-15\_v1** |
| ▶ |  | ■ | **S003\_GF\_2\_SAMPLE\_v1** |
| ∟ |  | ◆ | **S003\_GF\_2\_SAMPLE\_v1** |
| ▶ |  | ■ | **S004\_GF\_16S\_2\_v1** |
| ∟ |  | ◆ | **S004\_GF\_16S\_2\_v1** |

**If the lines for some categories do not extend all the way to the right end of the x-axis, that means that at least one of the samples in that category does not have that many sequences.**

  
  

|  |  |  |  |  |  |  |  |  |  |
| --- | --- | --- | --- | --- | --- | --- | --- | --- | --- |
| SampleID | Seqs/Sample | chao1 Ave. | chao1 Err. | observed\_species Ave. | observed\_species Err. | shannon Ave. | shannon Err. | simpson Ave. | simpson Err. |
| S001\_chic\_2\_sample\_16s\_10-23-15\_v2 | 10.0 | 15.450 | nan | 7.200 | nan | 2.643 | nan | 0.808 | nan || S001\_chic\_2\_sample\_16s\_10-23-15\_v2 | 21684.0 | 109.039 | nan | 104.900 | nan | 4.412 | nan | 0.904 | nan || S001\_chic\_2\_sample\_16s\_10-23-15\_v2 | 43358.0 | 111.619 | nan | 109.000 | nan | 4.417 | nan | 0.904 | nan || S001\_chic\_2\_sample\_16s\_10-23-15\_v2 | 65032.0 | 111.465 | nan | 110.500 | nan | 4.418 | nan | 0.904 | nan || S001\_chic\_2\_sample\_16s\_10-23-15\_v2 | 86706.0 | 111.183 | nan | 110.700 | nan | 4.419 | nan | 0.904 | nan || S001\_chic\_2\_sample\_16s\_10-23-15\_v2 | 108380.0 | 111.000 | nan | 110.900 | nan | 4.414 | nan | 0.904 | nan || S001\_chic\_2\_sample\_16s\_10-23-15\_v2 | 130054.0 | 111.000 | nan | 111.000 | nan | 4.419 | nan | 0.904 | nan || S001\_chic\_2\_sample\_16s\_10-23-15\_v2 | 151728.0 | 111.000 | nan | 111.000 | nan | 4.418 | nan | 0.904 | nan || S001\_chic\_2\_sample\_16s\_10-23-15\_v2 | 173402.0 | 111.000 | nan | 111.000 | nan | 4.417 | nan | 0.904 | nan || S001\_chic\_2\_sample\_16s\_10-23-15\_v2 | 195076.0 | 111.000 | nan | 111.000 | nan | 4.417 | nan | 0.904 | nan || S001\_chic\_2\_sample\_16s\_10-23-15\_v2 | 216750.0 | 111.000 | nan | 111.000 | nan | 4.418 | nan | 0.904 | nan || S002\_Sarayu\_Chic\_16s\_2-Sample\_10-30-15\_v1 | 10.0 | 18.733 | nan | 6.800 | nan | 2.549 | nan | 0.798 | nan || S002\_Sarayu\_Chic\_16s\_2-Sample\_10-30-15\_v1 | 21684.0 | 105.707 | nan | 102.100 | nan | 4.377 | nan | 0.901 | nan || S002\_Sarayu\_Chic\_16s\_2-Sample\_10-30-15\_v1 | 43358.0 | 108.013 | nan | 105.700 | nan | 4.380 | nan | 0.901 | nan || S002\_Sarayu\_Chic\_16s\_2-Sample\_10-30-15\_v1 | 65032.0 | 107.233 | nan | 106.900 | nan | 4.375 | nan | 0.900 | nan || S002\_Sarayu\_Chic\_16s\_2-Sample\_10-30-15\_v1 | 86706.0 | 107.933 | nan | 107.900 | nan | 4.381 | nan | 0.901 | nan || S002\_Sarayu\_Chic\_16s\_2-Sample\_10-30-15\_v1 | 108380.0 | 107.900 | nan | 107.900 | nan | 4.381 | nan | 0.901 | nan || S002\_Sarayu\_Chic\_16s\_2-Sample\_10-30-15\_v1 | 130054.0 | 108.000 | nan | 108.000 | nan | 4.380 | nan | 0.901 | nan || S002\_Sarayu\_Chic\_16s\_2-Sample\_10-30-15\_v1 | 151728.0 | 108.000 | nan | 108.000 | nan | 4.382 | nan | 0.901 | nan || S002\_Sarayu\_Chic\_16s\_2-Sample\_10-30-15\_v1 | 173402.0 | 108.000 | nan | 108.000 | nan | 4.380 | nan | 0.901 | nan || S002\_Sarayu\_Chic\_16s\_2-Sample\_10-30-15\_v1 | 195076.0 | 108.000 | nan | 108.000 | nan | 4.382 | nan | 0.901 | nan || S002\_Sarayu\_Chic\_16s\_2-Sample\_10-30-15\_v1 | 216750.0 | 108.000 | nan | 108.000 | nan | 4.380 | nan | 0.901 | nan || S003\_GF\_2\_SAMPLE\_v1 | 10.0 | 17.600 | nan | 7.500 | nan | 2.739 | nan | 0.826 | nan || S003\_GF\_2\_SAMPLE\_v1 | 21684.0 | 85.239 | nan | 84.700 | nan | 4.622 | nan | 0.937 | nan || S003\_GF\_2\_SAMPLE\_v1 | 43358.0 | 85.900 | nan | 85.900 | nan | 4.619 | nan | 0.936 | nan || S003\_GF\_2\_SAMPLE\_v1 | 65032.0 | 86.000 | nan | 86.000 | nan | 4.622 | nan | 0.936 | nan || S003\_GF\_2\_SAMPLE\_v1 | 86706.0 | 86.000 | nan | 86.000 | nan | 4.619 | nan | 0.936 | nan || S003\_GF\_2\_SAMPLE\_v1 | 108380.0 | 86.000 | nan | 86.000 | nan | 4.621 | nan | 0.936 | nan || S003\_GF\_2\_SAMPLE\_v1 | 130054.0 | nan | nan | nan | nan | nan | nan | nan | nan || S003\_GF\_2\_SAMPLE\_v1 | 151728.0 | nan | nan | nan | nan | nan | nan | nan | nan || S003\_GF\_2\_SAMPLE\_v1 | 173402.0 | nan | nan | nan | nan | nan | nan | nan | nan || S003\_GF\_2\_SAMPLE\_v1 | 195076.0 | nan | nan | nan | nan | nan | nan | nan | nan || S003\_GF\_2\_SAMPLE\_v1 | 216750.0 | nan | nan | nan | nan | nan | nan | nan | nan || S004\_GF\_16S\_2\_v1 | 10.0 | 8.200 | nan | 5.700 | nan | 2.252 | nan | 0.744 | nan || S004\_GF\_16S\_2\_v1 | 21684.0 | nan | nan | nan | nan | nan | nan | nan | nan || S004\_GF\_16S\_2\_v1 | 43358.0 | nan | nan | nan | nan | nan | nan | nan | nan || S004\_GF\_16S\_2\_v1 | 65032.0 | nan | nan | nan | nan | nan | nan | nan | nan || S004\_GF\_16S\_2\_v1 | 86706.0 | nan | nan | nan | nan | nan | nan | nan | nan || S004\_GF\_16S\_2\_v1 | 108380.0 | nan | nan | nan | nan | nan | nan | nan | nan || S004\_GF\_16S\_2\_v1 | 130054.0 | nan | nan | nan | nan | nan | nan | nan | nan || S004\_GF\_16S\_2\_v1 | 151728.0 | nan | nan | nan | nan | nan | nan | nan | nan || S004\_GF\_16S\_2\_v1 | 173402.0 | nan | nan | nan | nan | nan | nan | nan | nan || S004\_GF\_16S\_2\_v1 | 195076.0 | nan | nan | nan | nan | nan | nan | nan | nan || S004\_GF\_16S\_2\_v1 | 216750.0 | nan | nan | nan | nan | nan | nan | nan | nan |
