# Supplementary material for: A comparative analysis of microbial profile of Guinea fowl and chicken using metagenomic approach
Source: PLoS One. 2018 Mar 1;13(3):e0191029. doi: 10.1371/journal.pone.0191029 (PMC5832216; doi:10.1371/journal.pone.0191029)
Supplement: S1 File — 16SrRNA sequencing data revealing Intestinal microbial profile of the chicken gastrointestinal tract. (ZIP) [file pone.0191029.s002.zip › exports/alphaDiversityDir_genus/alpha_rarefaction_plots/rarefaction_plots.html]

Rarefaction Curves


|  |  |  |  |
| --- | --- | --- | --- |
| **Select a Metric:** | chao1 observed\_species shannon simpson | **Select a Category:** | SampleID |

  

**Show Categories:
 
All
None
Invert**

**Legend**

|  |  |  |  |
| --- | --- | --- | --- |
| ▶ |  | ■ | **S001\_chic\_2\_sample\_16s\_10-23-15\_v2** |
| ∟ |  | ◆ | **S001\_chic\_2\_sample\_16s\_10-23-15\_v2** |
| ▶ |  | ■ | **S002\_Sarayu\_Chic\_16s\_2-Sample\_10-30-15\_v1** |
| ∟ |  | ◆ | **S002\_Sarayu\_Chic\_16s\_2-Sample\_10-30-15\_v1** |
| ▶ |  | ■ | **S003\_GF\_2\_SAMPLE\_v1** |
| ∟ |  | ◆ | **S003\_GF\_2\_SAMPLE\_v1** |
| ▶ |  | ■ | **S004\_GF\_16S\_2\_v1** |
| ∟ |  | ◆ | **S004\_GF\_16S\_2\_v1** |
| ▶ |  | ■ | **S001\_chic\_2\_sample\_16s\_10-23-15\_v2** |
| ∟ |  | ◆ | **S001\_chic\_2\_sample\_16s\_10-23-15\_v2** |
| ▶ |  | ■ | **S002\_Sarayu\_Chic\_16s\_2-Sample\_10-30-15\_v1** |
| ∟ |  | ◆ | **S002\_Sarayu\_Chic\_16s\_2-Sample\_10-30-15\_v1** |
| ▶ |  | ■ | **S003\_GF\_2\_SAMPLE\_v1** |
| ∟ |  | ◆ | **S003\_GF\_2\_SAMPLE\_v1** |
| ▶ |  | ■ | **S004\_GF\_16S\_2\_v1** |
| ∟ |  | ◆ | **S004\_GF\_16S\_2\_v1** |
| ▶ |  | ■ | **S001\_chic\_2\_sample\_16s\_10-23-15\_v2** |
| ∟ |  | ◆ | **S001\_chic\_2\_sample\_16s\_10-23-15\_v2** |
| ▶ |  | ■ | **S002\_Sarayu\_Chic\_16s\_2-Sample\_10-30-15\_v1** |
| ∟ |  | ◆ | **S002\_Sarayu\_Chic\_16s\_2-Sample\_10-30-15\_v1** |
| ▶ |  | ■ | **S003\_GF\_2\_SAMPLE\_v1** |
| ∟ |  | ◆ | **S003\_GF\_2\_SAMPLE\_v1** |
| ▶ |  | ■ | **S004\_GF\_16S\_2\_v1** |
| ∟ |  | ◆ | **S004\_GF\_16S\_2\_v1** |
| ▶ |  | ■ | **S001\_chic\_2\_sample\_16s\_10-23-15\_v2** |
| ∟ |  | ◆ | **S001\_chic\_2\_sample\_16s\_10-23-15\_v2** |
| ▶ |  | ■ | **S002\_Sarayu\_Chic\_16s\_2-Sample\_10-30-15\_v1** |
| ∟ |  | ◆ | **S002\_Sarayu\_Chic\_16s\_2-Sample\_10-30-15\_v1** |
| ▶ |  | ■ | **S003\_GF\_2\_SAMPLE\_v1** |
| ∟ |  | ◆ | **S003\_GF\_2\_SAMPLE\_v1** |
| ▶ |  | ■ | **S004\_GF\_16S\_2\_v1** |
| ∟ |  | ◆ | **S004\_GF\_16S\_2\_v1** |

**If the lines for some categories do not extend all the way to the right end of the x-axis, that means that at least one of the samples in that category does not have that many sequences.**

  
  

|  |  |  |  |  |  |  |  |  |  |
| --- | --- | --- | --- | --- | --- | --- | --- | --- | --- |
| SampleID | Seqs/Sample | chao1 Ave. | chao1 Err. | observed\_species Ave. | observed\_species Err. | shannon Ave. | shannon Err. | simpson Ave. | simpson Err. |
| S001\_chic\_2\_sample\_16s\_10-23-15\_v2 | 10.0 | 14.900 | nan | 6.700 | nan | 2.529 | nan | 0.796 | nan || S001\_chic\_2\_sample\_16s\_10-23-15\_v2 | 37144.0 | 76.462 | nan | 73.200 | nan | 3.792 | nan | 0.883 | nan || S001\_chic\_2\_sample\_16s\_10-23-15\_v2 | 74278.0 | 78.575 | nan | 76.900 | nan | 3.798 | nan | 0.884 | nan || S001\_chic\_2\_sample\_16s\_10-23-15\_v2 | 111412.0 | 77.742 | nan | 77.300 | nan | 3.794 | nan | 0.884 | nan || S001\_chic\_2\_sample\_16s\_10-23-15\_v2 | 148546.0 | 78.050 | nan | 77.900 | nan | 3.796 | nan | 0.884 | nan || S001\_chic\_2\_sample\_16s\_10-23-15\_v2 | 185680.0 | 78.000 | nan | 77.900 | nan | 3.797 | nan | 0.884 | nan || S001\_chic\_2\_sample\_16s\_10-23-15\_v2 | 222814.0 | 78.000 | nan | 78.000 | nan | 3.797 | nan | 0.884 | nan || S001\_chic\_2\_sample\_16s\_10-23-15\_v2 | 259948.0 | 78.000 | nan | 78.000 | nan | 3.796 | nan | 0.884 | nan || S001\_chic\_2\_sample\_16s\_10-23-15\_v2 | 297082.0 | 78.000 | nan | 78.000 | nan | 3.795 | nan | 0.884 | nan || S001\_chic\_2\_sample\_16s\_10-23-15\_v2 | 334216.0 | 78.000 | nan | 78.000 | nan | 3.794 | nan | 0.883 | nan || S001\_chic\_2\_sample\_16s\_10-23-15\_v2 | 371350.0 | 78.000 | nan | 78.000 | nan | 3.795 | nan | 0.884 | nan || S002\_Sarayu\_Chic\_16s\_2-Sample\_10-30-15\_v1 | 10.0 | 10.900 | nan | 6.900 | nan | 2.608 | nan | 0.810 | nan || S002\_Sarayu\_Chic\_16s\_2-Sample\_10-30-15\_v1 | 37144.0 | 74.475 | nan | 70.600 | nan | 3.748 | nan | 0.879 | nan || S002\_Sarayu\_Chic\_16s\_2-Sample\_10-30-15\_v1 | 74278.0 | 75.157 | nan | 74.400 | nan | 3.747 | nan | 0.879 | nan || S002\_Sarayu\_Chic\_16s\_2-Sample\_10-30-15\_v1 | 111412.0 | 76.267 | nan | 75.500 | nan | 3.746 | nan | 0.879 | nan || S002\_Sarayu\_Chic\_16s\_2-Sample\_10-30-15\_v1 | 148546.0 | 75.850 | nan | 75.800 | nan | 3.747 | nan | 0.879 | nan || S002\_Sarayu\_Chic\_16s\_2-Sample\_10-30-15\_v1 | 185680.0 | 75.900 | nan | 75.900 | nan | 3.749 | nan | 0.879 | nan || S002\_Sarayu\_Chic\_16s\_2-Sample\_10-30-15\_v1 | 222814.0 | 76.000 | nan | 76.000 | nan | 3.747 | nan | 0.879 | nan || S002\_Sarayu\_Chic\_16s\_2-Sample\_10-30-15\_v1 | 259948.0 | 76.000 | nan | 76.000 | nan | 3.747 | nan | 0.879 | nan || S002\_Sarayu\_Chic\_16s\_2-Sample\_10-30-15\_v1 | 297082.0 | 76.000 | nan | 76.000 | nan | 3.746 | nan | 0.879 | nan || S002\_Sarayu\_Chic\_16s\_2-Sample\_10-30-15\_v1 | 334216.0 | 76.000 | nan | 76.000 | nan | 3.747 | nan | 0.879 | nan || S002\_Sarayu\_Chic\_16s\_2-Sample\_10-30-15\_v1 | 371350.0 | 76.000 | nan | 76.000 | nan | 3.747 | nan | 0.879 | nan || S003\_GF\_2\_SAMPLE\_v1 | 10.0 | 20.250 | nan | 7.500 | nan | 2.727 | nan | 0.822 | nan || S003\_GF\_2\_SAMPLE\_v1 | 37144.0 | 71.928 | nan | 71.000 | nan | 4.146 | nan | 0.905 | nan || S003\_GF\_2\_SAMPLE\_v1 | 74278.0 | 72.050 | nan | 72.000 | nan | 4.150 | nan | 0.905 | nan || S003\_GF\_2\_SAMPLE\_v1 | 111412.0 | 72.000 | nan | 72.000 | nan | 4.151 | nan | 0.905 | nan || S003\_GF\_2\_SAMPLE\_v1 | 148546.0 | 72.000 | nan | 72.000 | nan | 4.149 | nan | 0.905 | nan || S003\_GF\_2\_SAMPLE\_v1 | 185680.0 | 72.000 | nan | 72.000 | nan | 4.149 | nan | 0.905 | nan || S003\_GF\_2\_SAMPLE\_v1 | 222814.0 | 72.000 | nan | 72.000 | nan | 4.150 | nan | 0.905 | nan || S003\_GF\_2\_SAMPLE\_v1 | 259948.0 | nan | nan | nan | nan | nan | nan | nan | nan || S003\_GF\_2\_SAMPLE\_v1 | 297082.0 | nan | nan | nan | nan | nan | nan | nan | nan || S003\_GF\_2\_SAMPLE\_v1 | 334216.0 | nan | nan | nan | nan | nan | nan | nan | nan || S003\_GF\_2\_SAMPLE\_v1 | 371350.0 | nan | nan | nan | nan | nan | nan | nan | nan || S004\_GF\_16S\_2\_v1 | 10.0 | 9.183 | nan | 5.900 | nan | 2.326 | nan | 0.764 | nan || S004\_GF\_16S\_2\_v1 | 37144.0 | nan | nan | nan | nan | nan | nan | nan | nan || S004\_GF\_16S\_2\_v1 | 74278.0 | nan | nan | nan | nan | nan | nan | nan | nan || S004\_GF\_16S\_2\_v1 | 111412.0 | nan | nan | nan | nan | nan | nan | nan | nan || S004\_GF\_16S\_2\_v1 | 148546.0 | nan | nan | nan | nan | nan | nan | nan | nan || S004\_GF\_16S\_2\_v1 | 185680.0 | nan | nan | nan | nan | nan | nan | nan | nan || S004\_GF\_16S\_2\_v1 | 222814.0 | nan | nan | nan | nan | nan | nan | nan | nan || S004\_GF\_16S\_2\_v1 | 259948.0 | nan | nan | nan | nan | nan | nan | nan | nan || S004\_GF\_16S\_2\_v1 | 297082.0 | nan | nan | nan | nan | nan | nan | nan | nan || S004\_GF\_16S\_2\_v1 | 334216.0 | nan | nan | nan | nan | nan | nan | nan | nan || S004\_GF\_16S\_2\_v1 | 371350.0 | nan | nan | nan | nan | nan | nan | nan | nan |
