# Supplementary material for: A comparative analysis of microbial profile of Guinea fowl and chicken using metagenomic approach
Source: PLoS One. 2018 Mar 1;13(3):e0191029. doi: 10.1371/journal.pone.0191029 (PMC5832216; doi:10.1371/journal.pone.0191029)
Supplement: S1 File — 16SrRNA sequencing data revealing Intestinal microbial profile of the chicken gastrointestinal tract. (ZIP) [file pone.0191029.s002.zip › exports/alphaDiversityDir_family/alpha_rarefaction_plots/rarefaction_plots.html]

Rarefaction Curves


|  |  |  |  |
| --- | --- | --- | --- |
| **Select a Metric:** | chao1 observed\_species shannon simpson | **Select a Category:** | SampleID |

  

**Show Categories:
 
All
None
Invert**

**Legend**

|  |  |  |  |
| --- | --- | --- | --- |
| ▶ |  | ■ | **S001\_chic\_2\_sample\_16s\_10-23-15\_v2** |
| ∟ |  | ◆ | **S001\_chic\_2\_sample\_16s\_10-23-15\_v2** |
| ▶ |  | ■ | **S002\_Sarayu\_Chic\_16s\_2-Sample\_10-30-15\_v1** |
| ∟ |  | ◆ | **S002\_Sarayu\_Chic\_16s\_2-Sample\_10-30-15\_v1** |
| ▶ |  | ■ | **S003\_GF\_2\_SAMPLE\_v1** |
| ∟ |  | ◆ | **S003\_GF\_2\_SAMPLE\_v1** |
| ▶ |  | ■ | **S004\_GF\_16S\_2\_v1** |
| ∟ |  | ◆ | **S004\_GF\_16S\_2\_v1** |
| ▶ |  | ■ | **S001\_chic\_2\_sample\_16s\_10-23-15\_v2** |
| ∟ |  | ◆ | **S001\_chic\_2\_sample\_16s\_10-23-15\_v2** |
| ▶ |  | ■ | **S002\_Sarayu\_Chic\_16s\_2-Sample\_10-30-15\_v1** |
| ∟ |  | ◆ | **S002\_Sarayu\_Chic\_16s\_2-Sample\_10-30-15\_v1** |
| ▶ |  | ■ | **S003\_GF\_2\_SAMPLE\_v1** |
| ∟ |  | ◆ | **S003\_GF\_2\_SAMPLE\_v1** |
| ▶ |  | ■ | **S004\_GF\_16S\_2\_v1** |
| ∟ |  | ◆ | **S004\_GF\_16S\_2\_v1** |
| ▶ |  | ■ | **S001\_chic\_2\_sample\_16s\_10-23-15\_v2** |
| ∟ |  | ◆ | **S001\_chic\_2\_sample\_16s\_10-23-15\_v2** |
| ▶ |  | ■ | **S002\_Sarayu\_Chic\_16s\_2-Sample\_10-30-15\_v1** |
| ∟ |  | ◆ | **S002\_Sarayu\_Chic\_16s\_2-Sample\_10-30-15\_v1** |
| ▶ |  | ■ | **S003\_GF\_2\_SAMPLE\_v1** |
| ∟ |  | ◆ | **S003\_GF\_2\_SAMPLE\_v1** |
| ▶ |  | ■ | **S004\_GF\_16S\_2\_v1** |
| ∟ |  | ◆ | **S004\_GF\_16S\_2\_v1** |
| ▶ |  | ■ | **S001\_chic\_2\_sample\_16s\_10-23-15\_v2** |
| ∟ |  | ◆ | **S001\_chic\_2\_sample\_16s\_10-23-15\_v2** |
| ▶ |  | ■ | **S002\_Sarayu\_Chic\_16s\_2-Sample\_10-30-15\_v1** |
| ∟ |  | ◆ | **S002\_Sarayu\_Chic\_16s\_2-Sample\_10-30-15\_v1** |
| ▶ |  | ■ | **S003\_GF\_2\_SAMPLE\_v1** |
| ∟ |  | ◆ | **S003\_GF\_2\_SAMPLE\_v1** |
| ▶ |  | ■ | **S004\_GF\_16S\_2\_v1** |
| ∟ |  | ◆ | **S004\_GF\_16S\_2\_v1** |

**If the lines for some categories do not extend all the way to the right end of the x-axis, that means that at least one of the samples in that category does not have that many sequences.**

  
  

|  |  |  |  |  |  |  |  |  |  |
| --- | --- | --- | --- | --- | --- | --- | --- | --- | --- |
| SampleID | Seqs/Sample | chao1 Ave. | chao1 Err. | observed\_species Ave. | observed\_species Err. | shannon Ave. | shannon Err. | simpson Ave. | simpson Err. |
| S001\_chic\_2\_sample\_16s\_10-23-15\_v2 | 10.0 | 7.983 | nan | 5.500 | nan | 2.229 | nan | 0.752 | nan || S001\_chic\_2\_sample\_16s\_10-23-15\_v2 | 98756.0 | 78.720 | nan | 76.900 | nan | 3.374 | nan | 0.859 | nan || S001\_chic\_2\_sample\_16s\_10-23-15\_v2 | 197502.0 | 80.575 | nan | 80.200 | nan | 3.379 | nan | 0.859 | nan || S001\_chic\_2\_sample\_16s\_10-23-15\_v2 | 296248.0 | 81.083 | nan | 81.000 | nan | 3.377 | nan | 0.859 | nan || S001\_chic\_2\_sample\_16s\_10-23-15\_v2 | 394994.0 | 81.000 | nan | 81.000 | nan | 3.377 | nan | 0.859 | nan || S001\_chic\_2\_sample\_16s\_10-23-15\_v2 | 493740.0 | 81.000 | nan | 81.000 | nan | 3.376 | nan | 0.859 | nan || S001\_chic\_2\_sample\_16s\_10-23-15\_v2 | 592486.0 | 81.000 | nan | 81.000 | nan | 3.377 | nan | 0.859 | nan || S001\_chic\_2\_sample\_16s\_10-23-15\_v2 | 691232.0 | 81.000 | nan | 81.000 | nan | 3.377 | nan | 0.859 | nan || S001\_chic\_2\_sample\_16s\_10-23-15\_v2 | 789978.0 | 81.000 | nan | 81.000 | nan | 3.377 | nan | 0.859 | nan || S001\_chic\_2\_sample\_16s\_10-23-15\_v2 | 888724.0 | 81.000 | nan | 81.000 | nan | 3.377 | nan | 0.859 | nan || S001\_chic\_2\_sample\_16s\_10-23-15\_v2 | 987470.0 | 81.000 | nan | 81.000 | nan | 3.377 | nan | 0.859 | nan || S002\_Sarayu\_Chic\_16s\_2-Sample\_10-30-15\_v1 | 10.0 | 7.883 | nan | 5.700 | nan | 2.306 | nan | 0.770 | nan || S002\_Sarayu\_Chic\_16s\_2-Sample\_10-30-15\_v1 | 98756.0 | 78.945 | nan | 77.300 | nan | 3.378 | nan | 0.858 | nan || S002\_Sarayu\_Chic\_16s\_2-Sample\_10-30-15\_v1 | 197502.0 | 81.068 | nan | 80.400 | nan | 3.380 | nan | 0.858 | nan || S002\_Sarayu\_Chic\_16s\_2-Sample\_10-30-15\_v1 | 296248.0 | 80.600 | nan | 80.600 | nan | 3.379 | nan | 0.858 | nan || S002\_Sarayu\_Chic\_16s\_2-Sample\_10-30-15\_v1 | 394994.0 | 81.000 | nan | 81.000 | nan | 3.379 | nan | 0.858 | nan || S002\_Sarayu\_Chic\_16s\_2-Sample\_10-30-15\_v1 | 493740.0 | 81.000 | nan | 81.000 | nan | 3.379 | nan | 0.858 | nan || S002\_Sarayu\_Chic\_16s\_2-Sample\_10-30-15\_v1 | 592486.0 | 81.000 | nan | 81.000 | nan | 3.380 | nan | 0.858 | nan || S002\_Sarayu\_Chic\_16s\_2-Sample\_10-30-15\_v1 | 691232.0 | 81.000 | nan | 81.000 | nan | 3.379 | nan | 0.858 | nan || S002\_Sarayu\_Chic\_16s\_2-Sample\_10-30-15\_v1 | 789978.0 | 81.000 | nan | 81.000 | nan | 3.379 | nan | 0.858 | nan || S002\_Sarayu\_Chic\_16s\_2-Sample\_10-30-15\_v1 | 888724.0 | 81.000 | nan | 81.000 | nan | 3.379 | nan | 0.858 | nan || S002\_Sarayu\_Chic\_16s\_2-Sample\_10-30-15\_v1 | 987470.0 | 81.000 | nan | 81.000 | nan | 3.379 | nan | 0.858 | nan || S003\_GF\_2\_SAMPLE\_v1 | 10.0 | 23.183 | nan | 8.000 | nan | 2.887 | nan | 0.850 | nan || S003\_GF\_2\_SAMPLE\_v1 | 98756.0 | 100.207 | nan | 96.200 | nan | 4.365 | nan | 0.935 | nan || S003\_GF\_2\_SAMPLE\_v1 | 197502.0 | 102.785 | nan | 100.500 | nan | 4.365 | nan | 0.935 | nan || S003\_GF\_2\_SAMPLE\_v1 | 296248.0 | 101.975 | nan | 101.600 | nan | 4.364 | nan | 0.935 | nan || S003\_GF\_2\_SAMPLE\_v1 | 394994.0 | 102.000 | nan | 102.000 | nan | 4.364 | nan | 0.935 | nan || S003\_GF\_2\_SAMPLE\_v1 | 493740.0 | 102.000 | nan | 102.000 | nan | 4.365 | nan | 0.935 | nan || S003\_GF\_2\_SAMPLE\_v1 | 592486.0 | 102.000 | nan | 102.000 | nan | 4.365 | nan | 0.935 | nan || S003\_GF\_2\_SAMPLE\_v1 | 691232.0 | 102.000 | nan | 102.000 | nan | 4.365 | nan | 0.935 | nan || S003\_GF\_2\_SAMPLE\_v1 | 789978.0 | 102.000 | nan | 102.000 | nan | 4.364 | nan | 0.935 | nan || S003\_GF\_2\_SAMPLE\_v1 | 888724.0 | 102.000 | nan | 102.000 | nan | 4.365 | nan | 0.935 | nan || S003\_GF\_2\_SAMPLE\_v1 | 987470.0 | nan | nan | nan | nan | nan | nan | nan | nan || S004\_GF\_16S\_2\_v1 | 10.0 | 15.150 | nan | 7.400 | nan | 2.744 | nan | 0.832 | nan || S004\_GF\_16S\_2\_v1 | 98756.0 | nan | nan | nan | nan | nan | nan | nan | nan || S004\_GF\_16S\_2\_v1 | 197502.0 | nan | nan | nan | nan | nan | nan | nan | nan || S004\_GF\_16S\_2\_v1 | 296248.0 | nan | nan | nan | nan | nan | nan | nan | nan || S004\_GF\_16S\_2\_v1 | 394994.0 | nan | nan | nan | nan | nan | nan | nan | nan || S004\_GF\_16S\_2\_v1 | 493740.0 | nan | nan | nan | nan | nan | nan | nan | nan || S004\_GF\_16S\_2\_v1 | 592486.0 | nan | nan | nan | nan | nan | nan | nan | nan || S004\_GF\_16S\_2\_v1 | 691232.0 | nan | nan | nan | nan | nan | nan | nan | nan || S004\_GF\_16S\_2\_v1 | 789978.0 | nan | nan | nan | nan | nan | nan | nan | nan || S004\_GF\_16S\_2\_v1 | 888724.0 | nan | nan | nan | nan | nan | nan | nan | nan || S004\_GF\_16S\_2\_v1 | 987470.0 | nan | nan | nan | nan | nan | nan | nan | nan |
