# Supplementary material for: A comparative analysis of microbial profile of Guinea fowl and chicken using metagenomic approach
Source: PLoS One. 2018 Mar 1;13(3):e0191029. doi: 10.1371/journal.pone.0191029 (PMC5832216; doi:10.1371/journal.pone.0191029)
Supplement: S1 File — 16SrRNA sequencing data revealing Intestinal microbial profile of the chicken gastrointestinal tract. (ZIP) [file pone.0191029.s002.zip › exports/betaDiversityDir_species/euclidean_emperor_pcoa_plot/index.html]

Emperor


|  |
| --- |
|  |


# WebGL is not enabled!

Emperor's visualization framework is WebGL based, it seems that your system doesn't have this resource available. Here is what you can do:

**Chrome:** Type "chrome://flags/" into the address bar, then search for "Disable WebGL". Disable this option if you haven't already. *Note:* If you follow these steps and still don't see an image, go to "chrome://flags/" and then search for "Override software rendering list" and enable this option.

**Safari:** Open Safari's menu and select Preferences. Click on the advanced tab, and then check "Show Developer" menu. Then open the "Developer" menu and select "Enable WebGL".

**Firefox:** Go to Options through Firefox > Options or Tools > Options. Go to Advanced, then General. Check "Use hardware acceleration when available" and restart Firefox.

**Other browsers:** The only browsers that support WebGL are Chrome, Safari, and Firefox. Please switch to these browsers when using Emperor.

*Note:* Once you went through these changes, reload the page and it should work!

Sources: Instructions for Chrome and Safari, and Firefox

PCoA
Parallel

- Key
- Colors
- Visibility
- Scaling
- Labels
- Axes
- Animations
- Options

Filter

|  |
| --- |
|  |
|  |
| ---    Global Sphere Opacity |
| Invert Selected |

|  |
| --- |
|  |
|  |
| ---    Global Sphere Scale |

Samples Label Visibility
  
Label Opacity


|  |  |
| --- | --- |
|  | Master Label Color |

|  |
| --- |
|  |
| Speed |
| Gradient Category |
|  |
| Trajectory Category |
|  |
|  |

|  |  |
| --- | --- |
|  | Axes Labels Color |
|  | Axes Color |
|  | Background Color |
| Scale coords by percent explained        ---   Filename (only letters, numbers, ., - and \_):     Create legend   For a PNG, simply press 'ctrl+p'. | |
