# Supplementary figures and images for: A comparative analysis of microbial profile of Guinea fowl and chicken using metagenomic approach
Source: PLoS One. 2018 Mar 1;13(3):e0191029. doi: 10.1371/journal.pone.0191029 (PMC5832216; doi:10.1371/journal.pone.0191029)

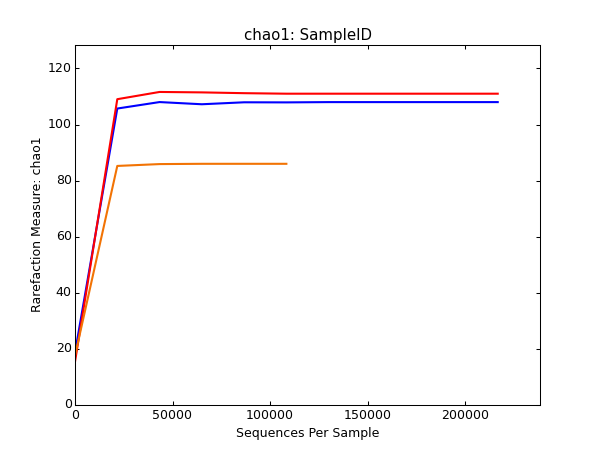

Supplement: S1 File — 16SrRNA sequencing data revealing Intestinal microbial profile of the chicken gastrointestinal tract. (ZIP) [file pone.0191029.s002.zip › exports/alphaDiversityDir_species/alpha_rarefaction_plots/average_plots/chao1SampleID.png]

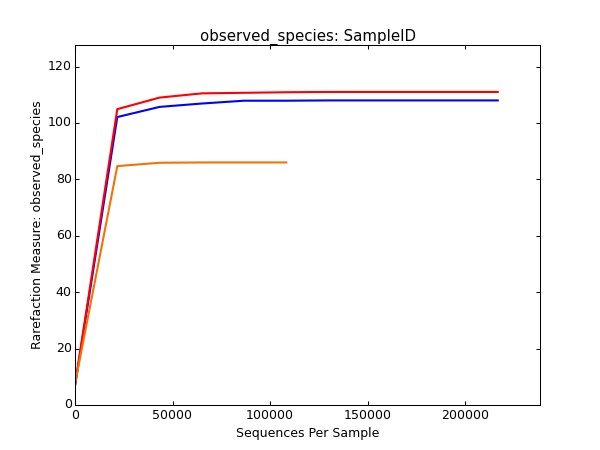

Supplement: S1 File — 16SrRNA sequencing data revealing Intestinal microbial profile of the chicken gastrointestinal tract. (ZIP) [file pone.0191029.s002.zip › exports/alphaDiversityDir_species/alpha_rarefaction_plots/average_plots/observed_speciesSampleID.png]

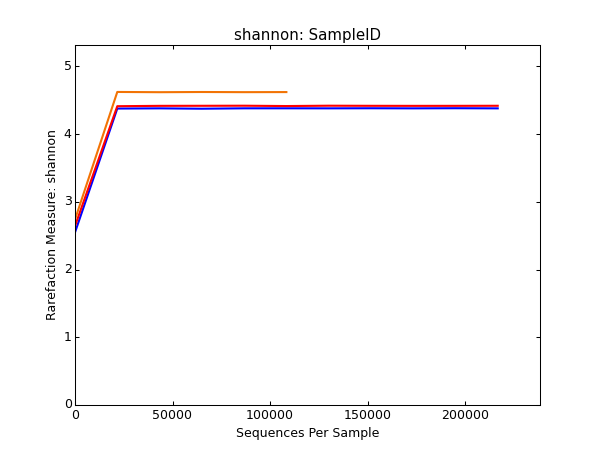

Supplement: S1 File — 16SrRNA sequencing data revealing Intestinal microbial profile of the chicken gastrointestinal tract. (ZIP) [file pone.0191029.s002.zip › exports/alphaDiversityDir_species/alpha_rarefaction_plots/average_plots/shannonSampleID.png]

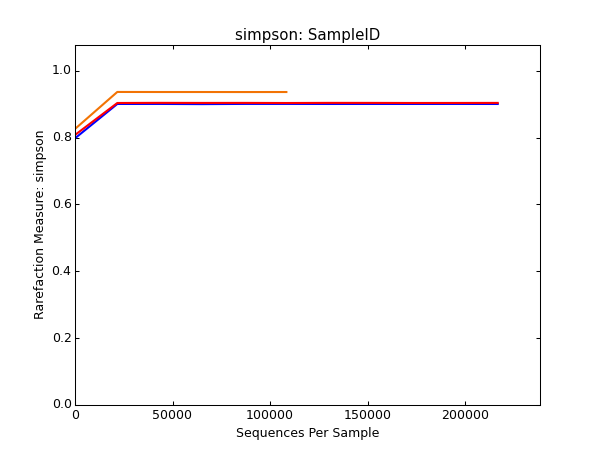

Supplement: S1 File — 16SrRNA sequencing data revealing Intestinal microbial profile of the chicken gastrointestinal tract. (ZIP) [file pone.0191029.s002.zip › exports/alphaDiversityDir_species/alpha_rarefaction_plots/average_plots/simpsonSampleID.png]

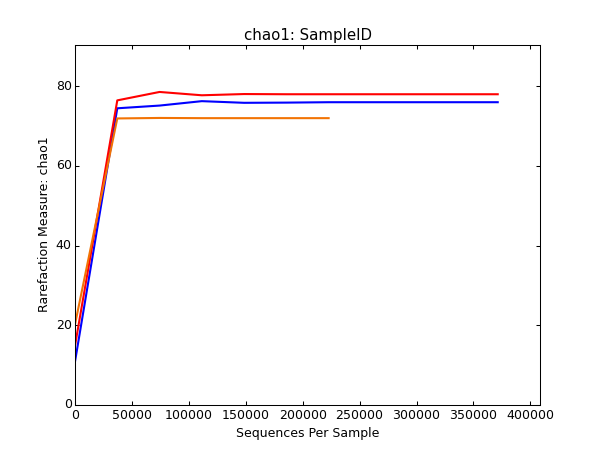

Supplement: S1 File — 16SrRNA sequencing data revealing Intestinal microbial profile of the chicken gastrointestinal tract. (ZIP) [file pone.0191029.s002.zip › exports/alphaDiversityDir_genus/alpha_rarefaction_plots/average_plots/chao1SampleID.png]

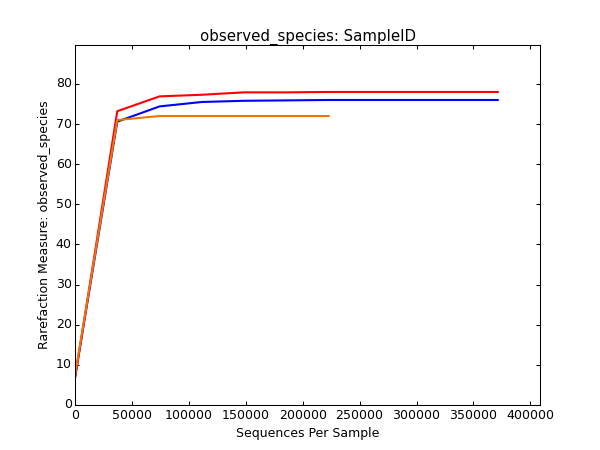

Supplement: S1 File — 16SrRNA sequencing data revealing Intestinal microbial profile of the chicken gastrointestinal tract. (ZIP) [file pone.0191029.s002.zip › exports/alphaDiversityDir_genus/alpha_rarefaction_plots/average_plots/observed_speciesSampleID.png]

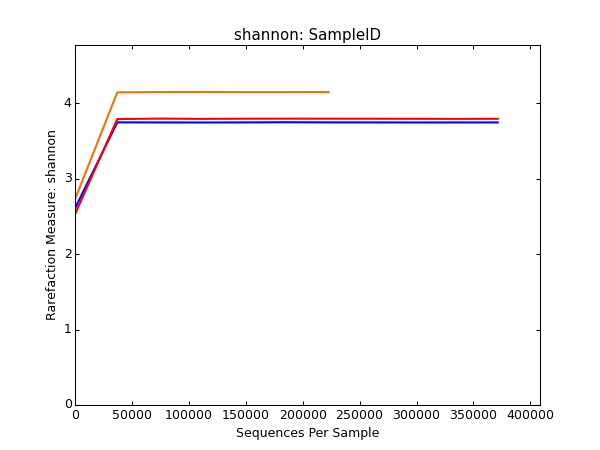

Supplement: S1 File — 16SrRNA sequencing data revealing Intestinal microbial profile of the chicken gastrointestinal tract. (ZIP) [file pone.0191029.s002.zip › exports/alphaDiversityDir_genus/alpha_rarefaction_plots/average_plots/shannonSampleID.png]

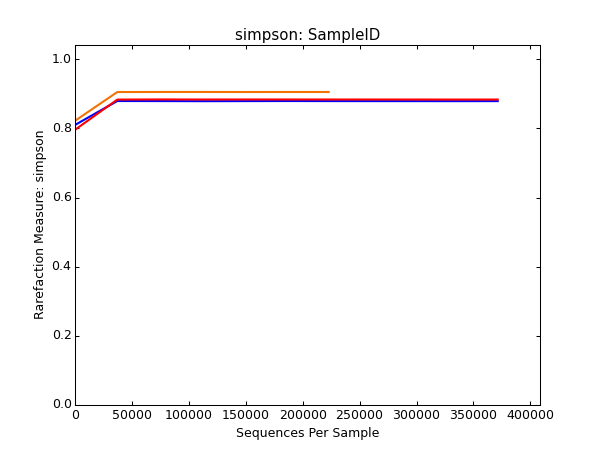

Supplement: S1 File — 16SrRNA sequencing data revealing Intestinal microbial profile of the chicken gastrointestinal tract. (ZIP) [file pone.0191029.s002.zip › exports/alphaDiversityDir_genus/alpha_rarefaction_plots/average_plots/simpsonSampleID.png]

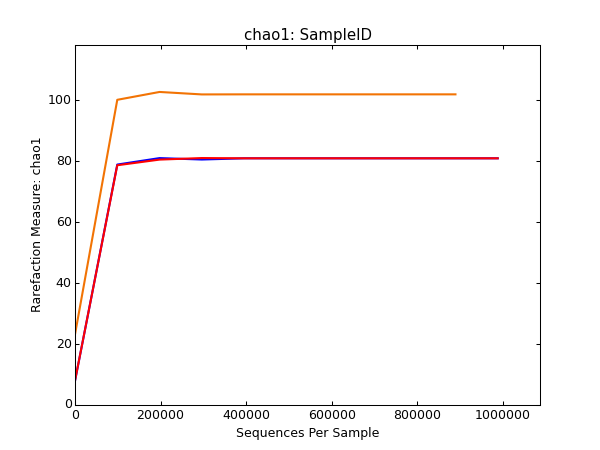

Supplement: S1 File — 16SrRNA sequencing data revealing Intestinal microbial profile of the chicken gastrointestinal tract. (ZIP) [file pone.0191029.s002.zip › exports/alphaDiversityDir_family/alpha_rarefaction_plots/average_plots/chao1SampleID.png]

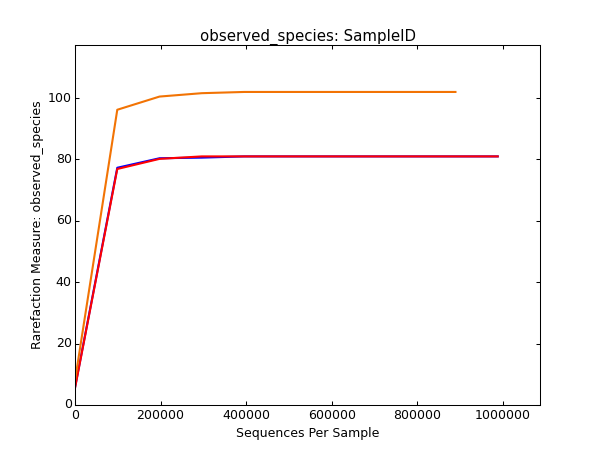

Supplement: S1 File — 16SrRNA sequencing data revealing Intestinal microbial profile of the chicken gastrointestinal tract. (ZIP) [file pone.0191029.s002.zip › exports/alphaDiversityDir_family/alpha_rarefaction_plots/average_plots/observed_speciesSampleID.png]

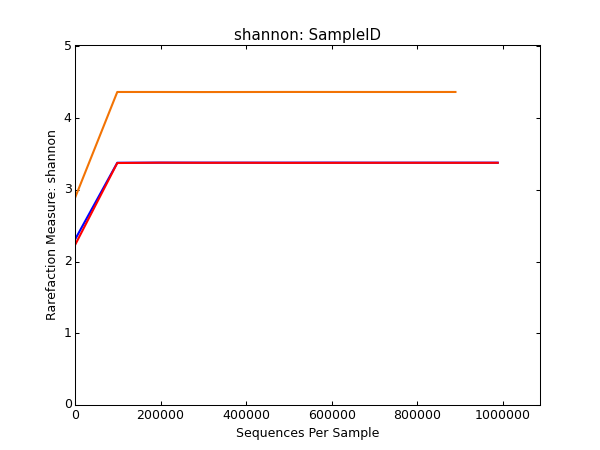

Supplement: S1 File — 16SrRNA sequencing data revealing Intestinal microbial profile of the chicken gastrointestinal tract. (ZIP) [file pone.0191029.s002.zip › exports/alphaDiversityDir_family/alpha_rarefaction_plots/average_plots/shannonSampleID.png]

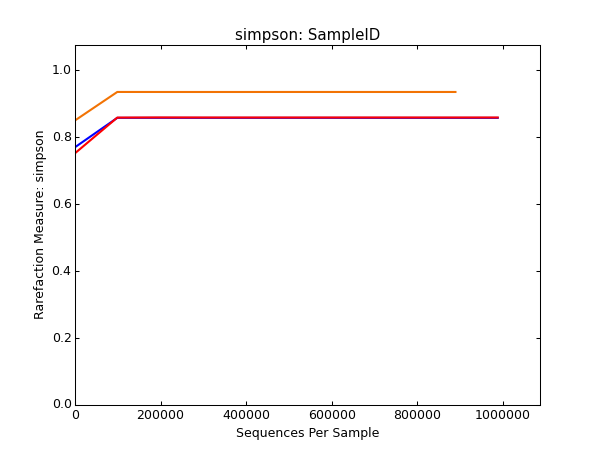

Supplement: S1 File — 16SrRNA sequencing data revealing Intestinal microbial profile of the chicken gastrointestinal tract. (ZIP) [file pone.0191029.s002.zip › exports/alphaDiversityDir_family/alpha_rarefaction_plots/average_plots/simpsonSampleID.png]

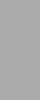

Supplement: S1 File — 16SrRNA sequencing data revealing Intestinal microbial profile of the chicken gastrointestinal tract. (ZIP) [file pone.0191029.s002.zip › exports/betaDiversityDir_species/euclidean_emperor_pcoa_plot/emperor_required_resources/css/images/ui-bg_flat_0_aaaaaa_40x100.png]

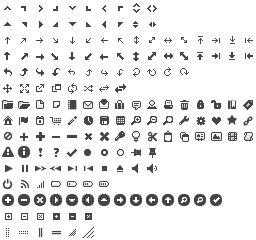

Supplement: S1 File — 16SrRNA sequencing data revealing Intestinal microbial profile of the chicken gastrointestinal tract. (ZIP) [file pone.0191029.s002.zip › exports/betaDiversityDir_species/euclidean_emperor_pcoa_plot/emperor_required_resources/css/images/ui-icons_454545_256x240.png]

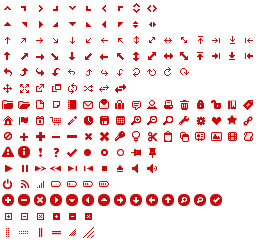

Supplement: S1 File — 16SrRNA sequencing data revealing Intestinal microbial profile of the chicken gastrointestinal tract. (ZIP) [file pone.0191029.s002.zip › exports/betaDiversityDir_species/euclidean_emperor_pcoa_plot/emperor_required_resources/css/images/ui-icons_cd0a0a_256x240.png]

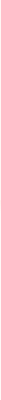

Supplement: S1 File — 16SrRNA sequencing data revealing Intestinal microbial profile of the chicken gastrointestinal tract. (ZIP) [file pone.0191029.s002.zip › exports/betaDiversityDir_species/euclidean_emperor_pcoa_plot/emperor_required_resources/css/images/ui-bg_glass_95_fef1ec_1x400.png]

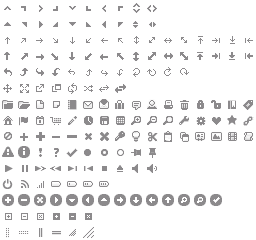

Supplement: S1 File — 16SrRNA sequencing data revealing Intestinal microbial profile of the chicken gastrointestinal tract. (ZIP) [file pone.0191029.s002.zip › exports/betaDiversityDir_species/euclidean_emperor_pcoa_plot/emperor_required_resources/css/images/ui-icons_888888_256x240.png]

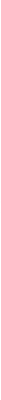

Supplement: S1 File — 16SrRNA sequencing data revealing Intestinal microbial profile of the chicken gastrointestinal tract. (ZIP) [file pone.0191029.s002.zip › exports/betaDiversityDir_species/euclidean_emperor_pcoa_plot/emperor_required_resources/css/images/ui-bg_glass_65_ffffff_1x400.png]

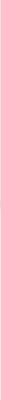

Supplement: S1 File — 16SrRNA sequencing data revealing Intestinal microbial profile of the chicken gastrointestinal tract. (ZIP) [file pone.0191029.s002.zip › exports/betaDiversityDir_species/euclidean_emperor_pcoa_plot/emperor_required_resources/css/images/ui-bg_glass_75_dadada_1x400.png]

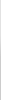

Supplement: S1 File — 16SrRNA sequencing data revealing Intestinal microbial profile of the chicken gastrointestinal tract. (ZIP) [file pone.0191029.s002.zip › exports/betaDiversityDir_species/euclidean_emperor_pcoa_plot/emperor_required_resources/css/images/ui-bg_highlight-soft_75_cccccc_1x100.png]

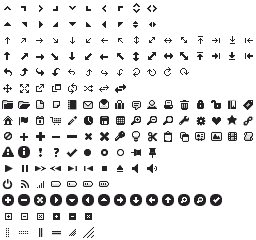

Supplement: S1 File — 16SrRNA sequencing data revealing Intestinal microbial profile of the chicken gastrointestinal tract. (ZIP) [file pone.0191029.s002.zip › exports/betaDiversityDir_species/euclidean_emperor_pcoa_plot/emperor_required_resources/css/images/ui-icons_222222_256x240.png]

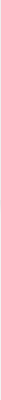

Supplement: S1 File — 16SrRNA sequencing data revealing Intestinal microbial profile of the chicken gastrointestinal tract. (ZIP) [file pone.0191029.s002.zip › exports/betaDiversityDir_species/euclidean_emperor_pcoa_plot/emperor_required_resources/css/images/ui-bg_glass_75_e6e6e6_1x400.png]

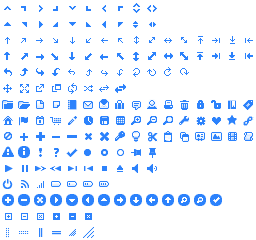

Supplement: S1 File — 16SrRNA sequencing data revealing Intestinal microbial profile of the chicken gastrointestinal tract. (ZIP) [file pone.0191029.s002.zip › exports/betaDiversityDir_species/euclidean_emperor_pcoa_plot/emperor_required_resources/css/images/ui-icons_2e83ff_256x240.png]

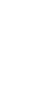

Supplement: S1 File — 16SrRNA sequencing data revealing Intestinal microbial profile of the chicken gastrointestinal tract. (ZIP) [file pone.0191029.s002.zip › exports/betaDiversityDir_species/euclidean_emperor_pcoa_plot/emperor_required_resources/css/images/ui-bg_flat_75_ffffff_40x100.png]

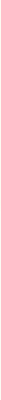

Supplement: S1 File — 16SrRNA sequencing data revealing Intestinal microbial profile of the chicken gastrointestinal tract. (ZIP) [file pone.0191029.s002.zip › exports/betaDiversityDir_species/euclidean_emperor_pcoa_plot/emperor_required_resources/css/images/ui-bg_glass_55_fbf9ee_1x400.png]

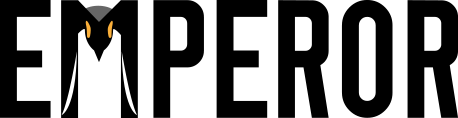

Supplement: S1 File — 16SrRNA sequencing data revealing Intestinal microbial profile of the chicken gastrointestinal tract. (ZIP) [file pone.0191029.s002.zip › exports/betaDiversityDir_species/euclidean_emperor_pcoa_plot/emperor_required_resources/img/emperor.png]

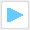

Supplement: S1 File — 16SrRNA sequencing data revealing Intestinal microbial profile of the chicken gastrointestinal tract. (ZIP) [file pone.0191029.s002.zip › exports/betaDiversityDir_species/euclidean_emperor_pcoa_plot/emperor_required_resources/img/play.png]

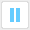

Supplement: S1 File — 16SrRNA sequencing data revealing Intestinal microbial profile of the chicken gastrointestinal tract. (ZIP) [file pone.0191029.s002.zip › exports/betaDiversityDir_species/euclidean_emperor_pcoa_plot/emperor_required_resources/img/pause.png]

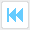

Supplement: S1 File — 16SrRNA sequencing data revealing Intestinal microbial profile of the chicken gastrointestinal tract. (ZIP) [file pone.0191029.s002.zip › exports/betaDiversityDir_species/euclidean_emperor_pcoa_plot/emperor_required_resources/img/reset.png]
